# A Schiff base complex of lanthanum on modified MCM-41 as a reusable nanocatalyst in the homoselective synthesis of 5-substituted 1*H*-tetrazoles†

**DOI:** 10.1039/d2ra05413b

**Published:** 2022-11-29

**Authors:** Bahman Tahmasbi, Mohsen Nikoorazm, Parisa Moradi, Yunes Abbasi Tyula

**Affiliations:** Department of Chemistry, Faculty of Science, Ilam University P. O. Box 69315516 Ilam Iran b.tahmasbi@ilam.ac.ir bah.tahmasbi@gmail.com m.nikorazm@ilam.ac.ir e_nikoorazm@yahoo.com

## Abstract

In this work, mesoporous MCM-41 was modified by a new Schiff-base formed from the condensation of triethylenetatramine and 5-bromosalicylaldehyde. Then, it was used for the stabilization of lanthanum metal (La-Schiff base@MCM-41) as a homoselective, reusable, efficient and biocompatible catalyst in the synthesis of 5-substituted 1*H*-tetrazole derivatives. The synthesized tetrazoles were characterized using ^1^H NMR and FT-IR spectroscopy and methods to measure their physical properties. La-Schiff base@MCM-41 was characterized by using various techniques such as ICP, CHN, XRD, TGA, BET, FT-IR spectroscopy, SEM, EDS and WDX. This catalyst has good stability and a heterogeneous nature, enabling it to be easily recovered and reused several times without significant loss in catalytic activity. This present strategy has important advantages such as utilizing PEG as a green solvent, short reaction times, excellent yields, easy recycling of the catalyst and pure separation of the products. The recovered La-Schiff base@MCM-41 catalyst was characterized by using FT-IR spectroscopy, SEM and AAS.

## Abstract 2

In this work, mesoporous MCM-41 was modified by a new Schiff-base formed from the condensation of triethylenetatramine and 5-bromosalicylaldehyde. Then, lanthanum was immobilized on its surface as reusable nanocatalyst in the synthesis of tetrazoles.

## Graphical abstract

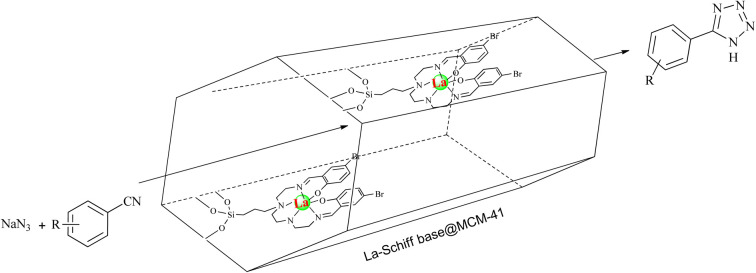

## 1 Introduction

Catalyst reusability is one of the principles of green chemistry and can increase the rate of a chemical process.^1–6^ Therefore, separation of used catalysts is one of the main challenges for chemists. On the other hand, a suitable, biocompatible and economical catalyst should have several properties such as simplicity of preparation, high catalytic activity, good selectivity, good stability, easy separation, and excellent reusability. The problems of recovering homogeneous catalysts and the low catalytic activity or low selectivity of heterogeneous catalysts have limited their application in industry and other sciences.^3,7–9^ To overcome these problems, nanomaterials or catalysts immobilized on nanomaterials seem to be ideal catalysts because nanomaterials have stability and a heterogeneous nature, enabling them to be easily recovered and reused like heterogeneous catalysts, and they also have high surface areas that can improve catalytic activity and selectivity in chemical procedures.^5,10^ Therefore, nanocatalysts exist at the border between heterogeneous and homogeneous catalysts because they have advantages from both, such as efficiency, selectivity and reusability.^5,10,11^ For example, various nanomaterials such as nanopolymers,^12^ carbon nanotubes,^13–15^ mesoporous silica,^16–18^ boehmite nanoparticles,^19–22^ graphene oxide nanosheets,^23^ biochar nanoparticles,^24,25^ magnetic nanoparticles,^7–9,26,27^ metal–organic frameworks^28^*etc.* have been employed in chemistry and especially in catalysis applications. Amongst the nanomaterials, macroporous or mesoporous nanomaterials especially MCM-41 have been widely used as an ideal support for immobilization of various catalysts and other applications.^29,30^ MCM-41 has also been used in other fields such as drug delivery,^31,32^ extraction,^33^ adsorption,^34,35^ sensors,^36,37^ supports for catalysts,^38–40^ and energy.^41^ This is because MCM-41 has unique properties, *e.g.* excellent thermal and chemical stability, high surface area (>1000 m^2^ g^−1^), easy surface functionalization, homogeneous hexagonal channel structure (1.5–10 nm pore diameters), large pore volumes (up to 1.3 ml g^−1^), good biocompatibility and easy separation from the reaction mixture.^38–43^ The large specific surface area of MCM-41 leads to a high capacity of catalyst loading. Also, the high thermal and chemical stability of MCM-41 allows the application of MCM-41 under harsh conditions and various chemical conditions. Moreover, the large pore volume of MCM-41 allows the application of MCM-41 in the immobilization of organic ligands and metal complexes into its channels.^38^ Therefore, we investigated a new Schiff-base complex of lanthanum-catalyst on MCM-41 (La-Schiff base@MCM-41) as an efficient, stable and recyclable nanocatalyst in the homoselective synthesis of 5-substituted 1*H*-tetrazole derivatives, because tetrazole derivatives have biological activity and they are also used as herbicides, analgesics, anti-HIV drug candidates, and antimicrobial, anti-inflammatory, anti-proliferative, and anticancer agents.^44–52^ For example, Candesartan, Irbesartan, Valsartan, Cilostazol, Losartan, Pranlukast and Pemiroplast are several pharmacologically important tetrazole derivatives.^53–55^

## 2 Experimental

### 2.1 Materials and instruments

The chemical compounds and solvents for synthesis of the catalyst and tetrazoles used in this study were purchased from Merck, Aldrich, Fluka or Iranian companies and used without any purification.

The size and morphology of the MCM-41, nanocatalyst and recovered catalyst were studied using FESEM imaging using a MIRA3TESCAN-XMU or FESEM-TESCAN MIRA3 Scanning Electron Microscope. Moreover, this Scanning Electron Microscope was employed to determine the elemental composition (EDS and WDX) of the nanocatalyst. Besides, the exact concentration of lanthanum in the catalysts was measured by an inductively coupled plasma instrument (ICP analysis) model ELAN 6100 DRC-e from PerkinElmer Company and AAS using a 400p-novAA instrument from Analytik Jena Company. The concentration of carbon, hydrogen and nitrogen in the nanocatalyst was measured by an elemental analyzer (CHN analysis) from the British company CQOSTECH. The TGA diagram of the nanocatalyst was recorded by a NETZSCH STA 449F3 Thermal Analysis device under air in the temperature range of 30–800 °C. Powder XRD patterns of the MCM-41 and nanocatalyst were recorded with CuKα radiation at 40 kV and 30 mA by a PW1730 instrument from Philips Company. FT-IR spectra of the nanomaterials and tetrazoles were recorded in KBr pellets using a VRTEX70 model Bruker FT-IR spectrometer. Nitrogen adsorption isotherms of MCM-41 and the nanocatalyst were obtained using a BELSORP MINI II device by a standard gas manifold at 77 K. In addition, the nanomaterial samples were degassed using a BEL PREP VAC II device before analysis at 120 °C for 2 h. NMR spectra of the tetrazoles were recorded using a Bruker DRX-400 spectrometer at 100–400 MHz. Melting points of the tetrazoles were obtained with an Electrothermal 9100 instrument.

### 2.2 Synthesis of the Schiff base ligand

**Scheme 1:**
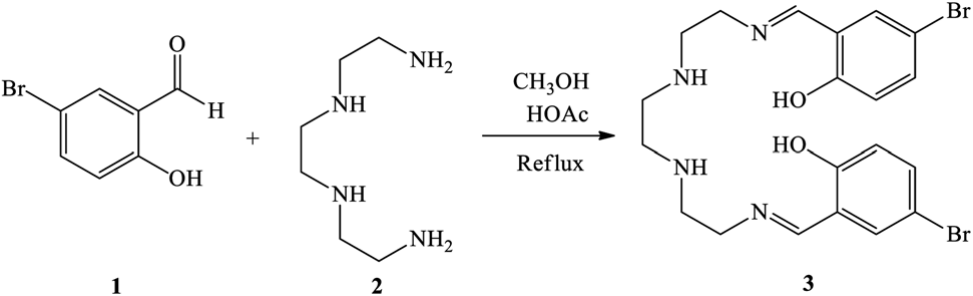
Synthesis of 2,5,8,11-tetraazadodeca-(1,11-diene-1,12-diyl)bis(4-bromophenol) as the Schiff base ligand (3).

First, 2,5,8,11-tetraazadodeca-1,11-diene-1,12-diyl)bis(4-bromophenol) as a new Schiff base ligand (3) was prepared by the condensation of 5-bromosalicylaldehyde (1) with triethylenetatramine (2) according to the illustrated procedure in Scheme 1. In this regard, a solution of triethylenetetramine (1 ml, 6.7 mmol) in methanol (30 ml) was added drop-wise to a solution of 5-bromosalicylaldehyde (2.693 g, 13.4 mmol) in methanol (20 ml) under stirring. Glacial acetic acid (4 drops) was also added to the reaction mixture and refluxed for 4 h. The resulting yellow precipitate was filtered off, washed with methanol, and dried at room temperature.

### 2.3 Preparation of the catalyst

**Scheme 2:**
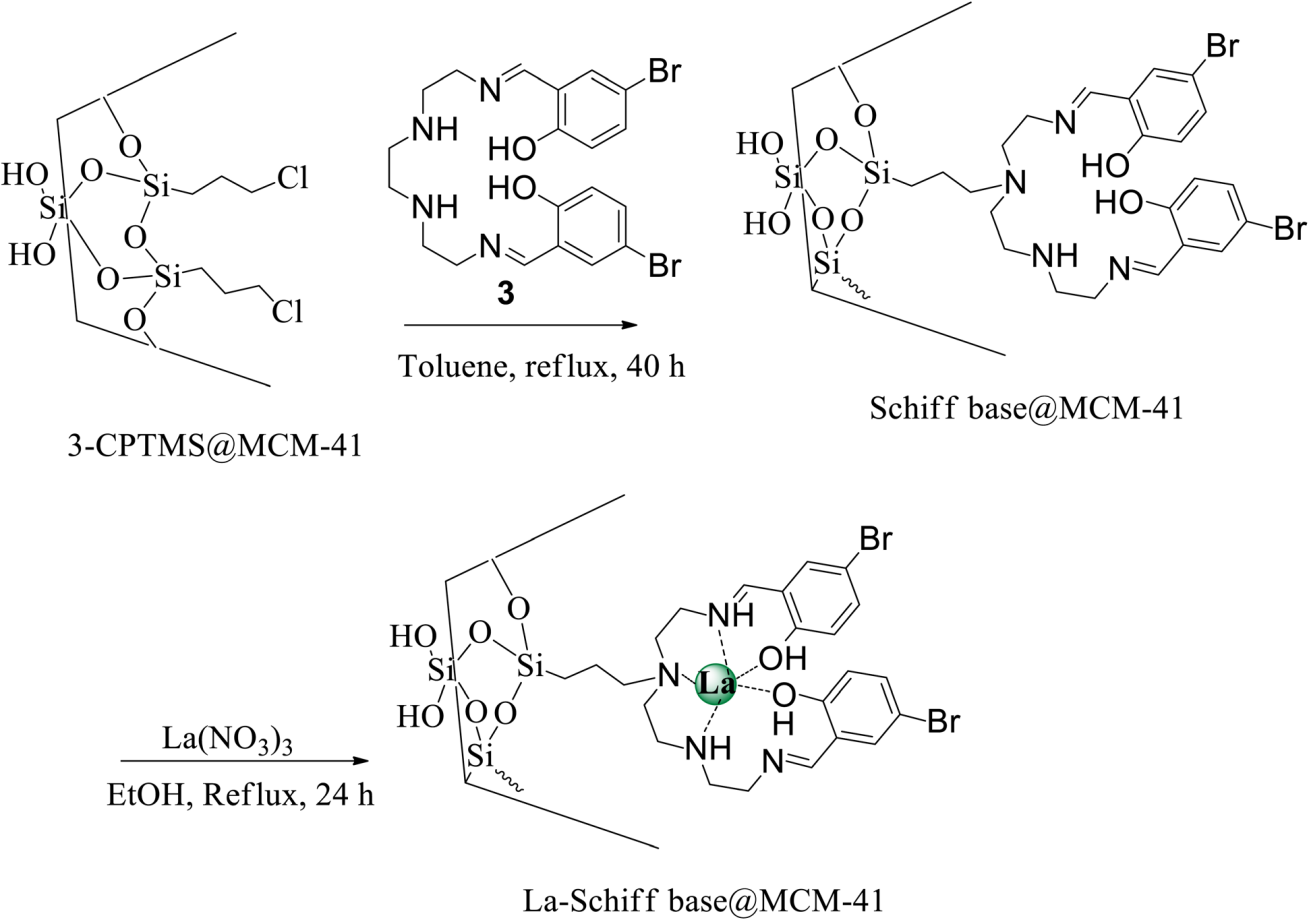
Synthesis of La-Schiff base@MCM-41.

The modified MCM-41 by (3-chloropropyl)triethoxysilane (3-CPTMS@MCM-41) was synthesized according to the reported procedure in the literature.^40^ To achieve immobilization of the Schiff base ligand (3) on MCM-41 (Schiff base@MCM-41), 1 g of 3-CPTMS@MCM-41 was refluxed with 3 (1 mmol) in toluene for 40 h. The obtained Schiff base@MCM-41 was isolated by simple filtration, washed with DMSO and hot ethanol and dried at room temperature. Finally, 1 g of Schiff base@MCM-41 was dispersed in ethanol, and then lanthanum nitrate (1 mmol) was added to the mixture and stirred for 24 h under reflux conditions. The obtained catalyst (La-Schiff base@MCM-41) was filtered, washed and dried at room temperature (Scheme 2).

### 2.4 General method for the synthesis of 5-substituted 1*H*-tetrazoles catalyzed by La-Schiff base@MCM-41

**Scheme 3:**
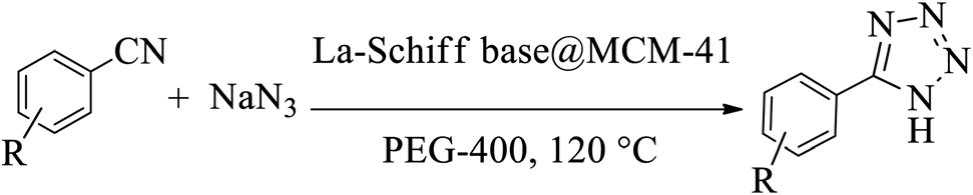
General method for the synthesis of 5-substituted 1*H*-tetrazoles in the presence of La-Schiff base@MCM-41.

The [3 + 2] cycloaddition of sodium azide salt (NaN_3_) with organic nitrile compounds was selected for the synthesis of heterocyclic tetrazoles in the presence of La-Schiff base@MCM-41 as catalyst. In this process, NaN_3_ (1.4 mmol) and nitrile compounds (1 mmol) were stirred in the presence of 50 mg of La-Schiff base@MCM-41 in PEG-400 at 120 °C. The reaction was monitored by using TLC. At the end of the reaction, the mixture was cooled down to room temperature. Then, the reaction mixture was diluted by water and ethyl acetate and the La-Schiff base@MCM-41 catalyst was separated using simple filtration. Finally, 10 ml of HCl (4 N) was added to the solution and the tetrazole products were extracted from ethyl acetate. The organic solvent was evaporated and the products were dried using anhydrous sodium sulfate (Scheme 3). The tetrazole products were obtained with yields of 89–98% and all obtained tetrazoles were confirmed by ^1^H NMR and FT-IR spectroscopy and physical property measurement such as melting point.

In order to show the reproducibility and reusability of the La-Schiff base@MCM-41 catalyst, the [3 + 2] cycloaddition of NaN_3_ with benzonitrile was repeated under optimized conditions. After completion of the reaction, the catalyst was recovered by centrifugation, washed with water and ethyl acetate and further employed in the next cycle. The La-Schiff base@MCM-41 catalyst showed good reproducibility and can reused up to 6 times.

### 2.5 Spectral data

#### 2.5.1 5-(4-Bromophenyl)-1*H*-tetrazole

^1^H NMR (400 MHz, DMSO): *δ*_H_ = 7.98–7.95 (d, *J* = 12 Hz, 2H), 7.83–7.81 (d, *J* = 12 Hz, 2H) ppm.

IR (KBr) cm^−1^: 3423, 3090, 3064, 2966, 2852, 2768, 1638, 1611, 1559, 1482, 1431, 1385, 1276, 1252, 1157, 1119, 1054, 1018, 988, 872, 828, 742, 614, 451.

#### 2.5.2 5-(3-Nitrophenyl)-1*H*-tetrazole

^1^H NMR (400 MHz, DMSO): *δ*_H_ = 18.44 (br, 1H), 8.82 (s, 1H), 8.48–8.45 (d, *J* = 12 Hz, 1H), 8.43–8.39 (d of q, *J* (d) = 12 Hz, *J* (q) = 2 Hz, 1H), 7.93–7.87 (t, *J* = 12 Hz, 1H) ppm.

#### 2.5.3 2-(1*H*-tetrazol-5-yl)benzonitrile

^1^H NMR (400 MHz, DMSO): *δ*_H_ = 8.10–8.06 (t, *J* = 8 Hz, 2H), 7.95–7.89 (t, *J* = 12 Hz, 1H), 7.80–7.75 (t, *J* = 12 Hz, 1H) ppm.

IR (KBr) cm^−1^: 3426, 3082, 3029, 2922, 2582, 2721, 2615, 2229, 2005, 1735, 1638, 1609, 1581, 1493, 1454, 1408, 1384, 1280, 1242, 1199,1167, 1122, 1100, 1066, 1047, 1113, 999, 970, 856, 783, 756, 724, 705, 665, 585, 555, 518, 500.

#### 2.5.4 5-(4-Nitrophenyl)-1*H*-tetrazole

^1^H NMR (400 MHz, DMSO): *δ*_H_ = 16.55 (br, 1H), 8.46–8.43 (d, *J* = 12 Hz, 2H), 8.31–8.28 (d, *J* = 12 Hz, 2H) ppm.

IR (KBr) cm^−1^: 3212, 3069, 2903, 1957, 1822, 1717, 1604, 1512, 1337, 1108, 1059, 991, 857, 773, 691, 491, 443.

#### 2.5.5 5-(4-Chlorophenyl)-1*H*-tetrazole

^1^H NMR (400 MHz, DMSO): *δ*_H_ = 16.99 (br, 1H), 8.06–8.03 (d, *J* = 12 Hz, 2H), 7.71–7.68 (d, *J* = 12 Hz, 2H) ppm.

IR (KBr) cm^−1^: 3420, 3096, 3070, 2922, 2852, 2733, 1638, 1611, 1487, 1458, 1434, 1385, 1277, 1254, 1160, 1121, 1096, 1052, 1019, 988, 878, 829, 743, 709, 693, 623, 506, 464.

#### 2.5.6 2-(1*H*-Tetrazol-5-yl)phenol

^1^H NMR (400 MHz, DMSO): *δ*_H_ = 8.00–7.98 (d, *J* = 12 Hz, 1H), 7.43–7.38 (t, *J* = 8 Hz, 1H), 7.08–7.06 (d, *J* = 8 Hz, 1H), 7.03–6.97 (t, *J* = 12 Hz, 1H) ppm.

IR (KBr) cm^−1^: 3431, 3252, 3062, 2930, 2686, 2567, 1737, 1610, 1546, 1477, 1391, 1356, 1295, 1232, 1156, 1112, 1086, 1002, 941, 809, 741, 542.

#### 2.5.7 5-(2-Chlorophenyl)-1*H*-tetrazole

^1^H NMR (400 MHz, DMSO): *δ*_H_ = 16.95 (br, 1H), 7.82–7.80 (d, *J* = 8 Hz, 1H), 7.73–7.70 (d, *J* = 12 Hz, 1H), 7.66–7.61 (t, *J* = 8 Hz, 1H), 7.58–7.54 (t, *J* = 8 Hz, 1H) ppm.

IR (KBr) cm^−1^: 3418, 2823, 2708, 1660, 1651, 1634, 1602, 1565, 1556, 1539, 1471, 1441, 1409, 1385, 1369, 1243, 1163, 1129, 1076, 1060, 1039, 1020, 1007, 988, 943, 874, 777, 747, 732, 651, 486, 453, 434.

#### 2.5.8 4-(1*H*-Tetrazol-5-yl)phenol

^1^H NMR (400 MHz, DMSO): *δ*_H_ = 16.51 (br, 1H), 10.15 (br, 1H), 7.87–7.84 (d, *J* = 12 Hz, 2H), 6.96–6.93 (d, *J* = 12 Hz, 1H) ppm.

#### 2.5.9 5-Phenyl-1*H*-tetrazole

^1^H NMR (400 MHz, DMSO): *δ*_H_ = 16.88 (br, 1H), 8.06–8.03 (m, 2H), 7.63–7.59 (m, 3H) ppm.

IR (KBr) cm^−1^: 3423, 3055, 2985, 2901, 2832, 1813, 1638, 1611, 1562, 1486, 1466, 1411, 1384, 1288, 1256, 1163, 18 084, 1057, 1034, 1015, 991, 959, 925, 784, 726, 703, 687, 619, 493, 462.

## 3 Results and discussion

### 3.1 Characterization of the catalyst

**Fig. 1:**
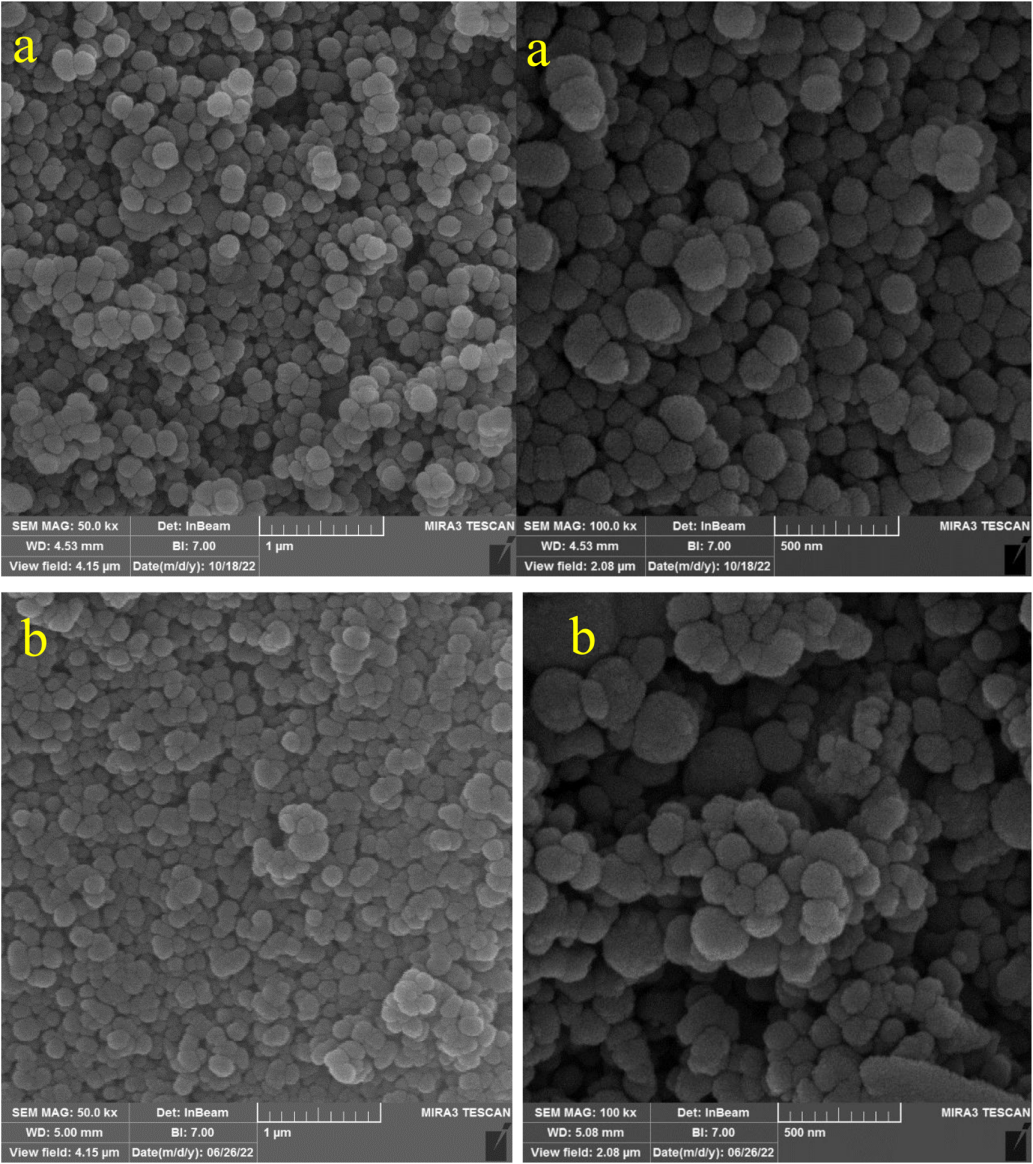
SEM images of (a) MCM-41 and (b) La-Schiff base@MCM-41 catalyst.

In the first step, MCM-41 with its surface modified by 3-chloropropyl)triethoxysilane was obtained based on a new reported procedure.^40^ Subsequently, 2,5,8,11-(tetraazadodeca-1,11-diene-1,12-diyl)bis(4-bromophenol), a new Schiff-base (ligand 3), complexed with lanthanum was stabilized on the chloro-modified MCM-41 nanoparticles (La-Schiff base@MCM-41) as an efficient and reusable nanocatalyst in the homoselective synthesis of 5-substituted 1*H*-tetrazole derivatives. This is the first report of immobilization of 2,5,8,11-tetraazadodeca-1,11-diene-1,12-diyl)bis(4-bromophenol) on MCM-41. Also, this is the first report where a lanthanum complex of 2,5,8,11-tetraazadodeca-1,11-diene-1,12-diyl)bis(4-bromophenol) was used as a catalyst in the synthesis of organic compounds. Therefore, this catalyst could be effective for other organic condensation or cycloaddition reactions. A FESEM-TESCAN MIRA III Scanning Electron Microscope instrument was employed to study the size and particle morphology of the MCM-41 and La-Schiff base@MCM-41 catalyst (Fig. 1). The obtained images show that MCM-41 and La-Schiff base@MCM-41 formed as uniform spherical shaped particles with quite homogeneous diameters of less than 100 nm. As can be seen in the SEM images, there is no significant difference in the shape and size of the MCM-41 particles and the catalyst, which indicates that the nanoparticles are stable during the modification.

**Fig. 2:**
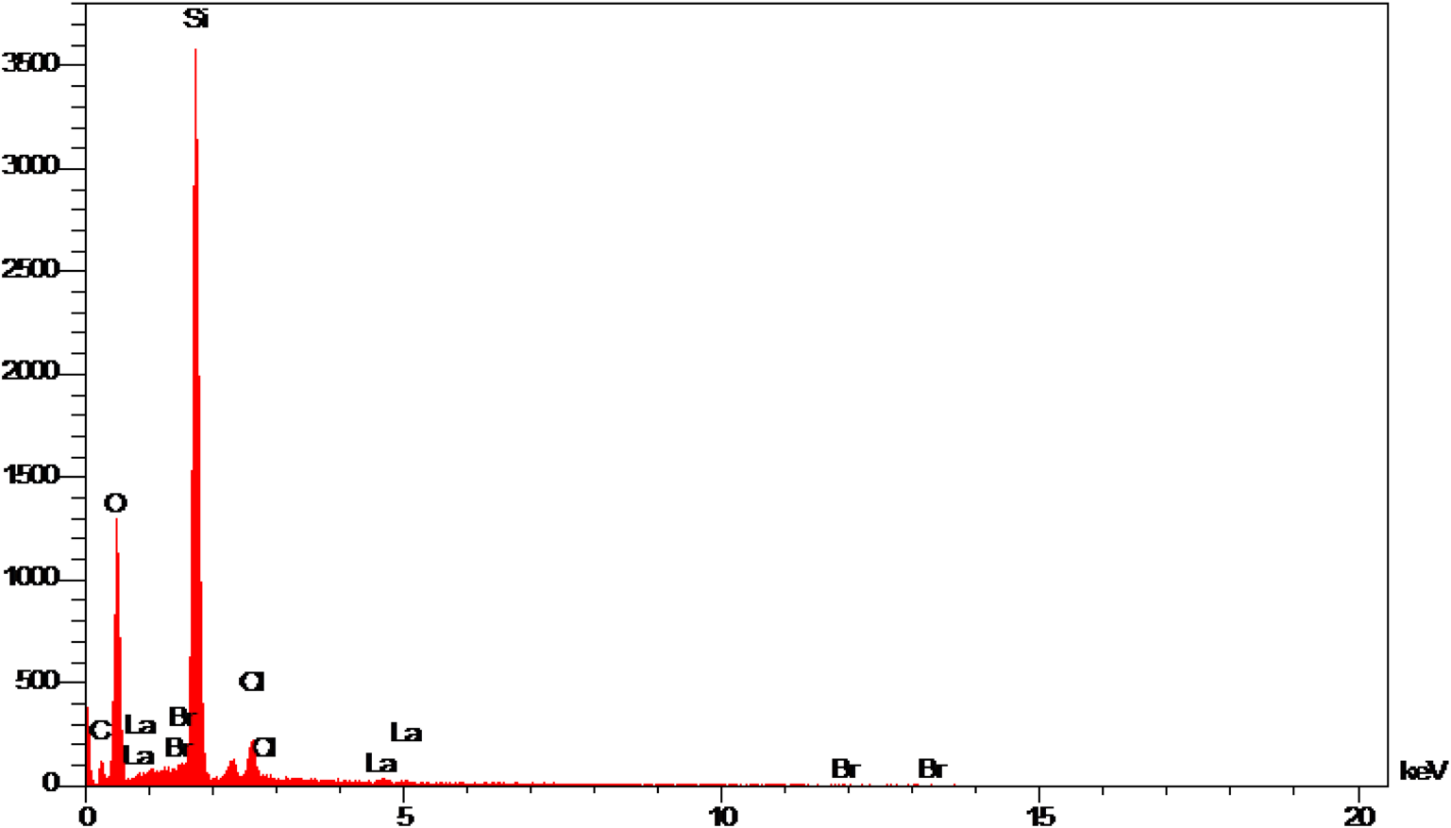
EDS spectrum of the La-Schiff base@MCM-41 catalyst.

**Fig. 3:**
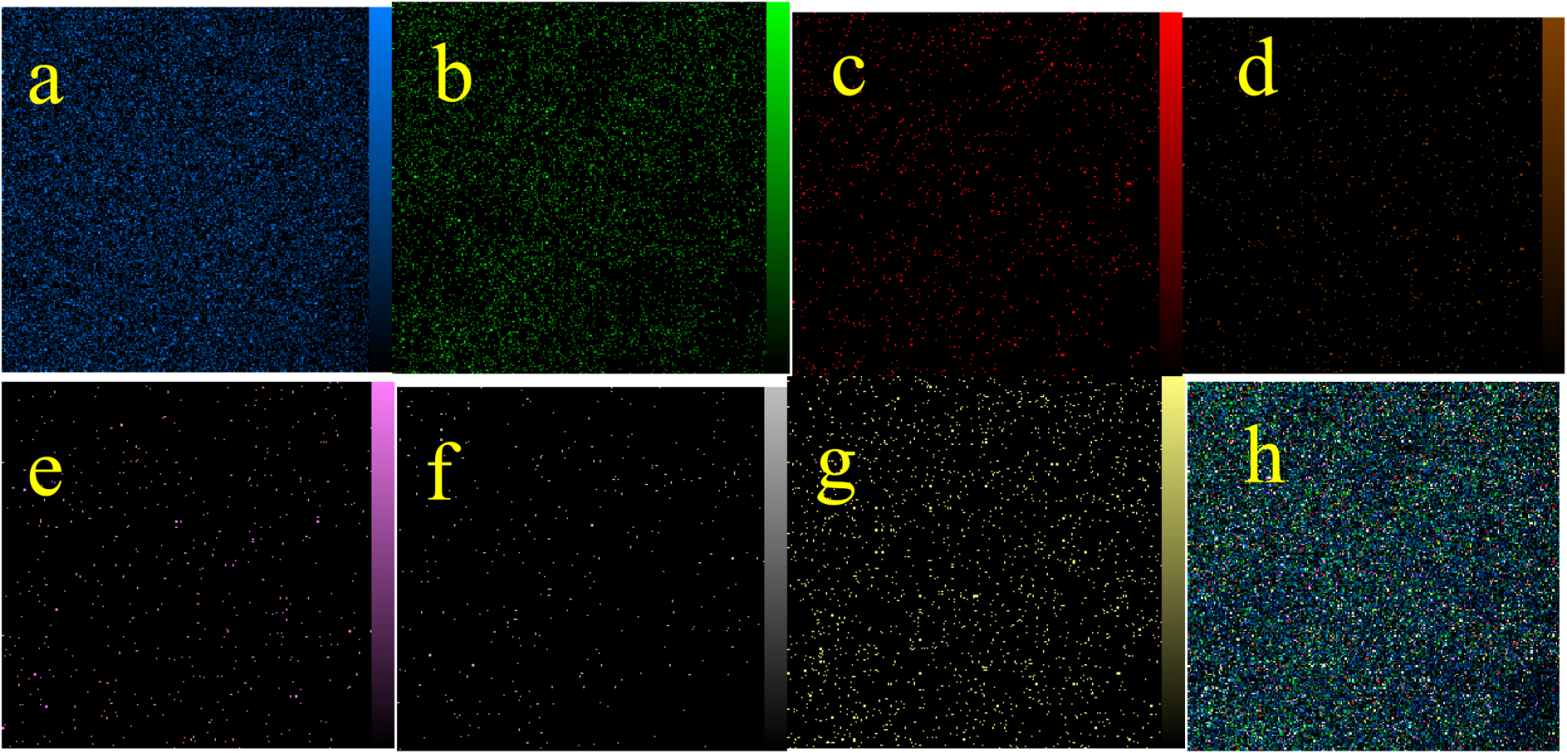
Elemental mapping of (a) Si, (b) O, (c) C, (d) Br, (e) La, (f) N, and (g) Cl and (h) the combination of all elements for the La-Schiff base@MCM-41 catalyst.

The qualitative elemental composition analysis of the La-Schiff base@MCM-41 catalyst was performed using energy-dispersive X-ray spectroscopy (EDS) using a FESEM-TESCAN MIRA III Scanning Electron Microscope instrument (Fig. 2). The EDS diagram contains the special energy (keV) of each element in terms of intensity. A quantitative increase in the amount of each element leads to an increase in the intensity of its peak in the EDS diagram. As shown in the EDS diagram of the La-Schiff base@MCM-41 catalyst, silicon and oxygen elements have the highest intensity, which are related to the skeleton of MCM-41. Also, the EDS diagram shows the presence of C, N and Br elements in the structure of the La-Schiff base@MCM-41 catalyst, which shows that the Schiff-base ligand is well stabilized on the MCM-41. Also, the EDS diagram shows La element in the structure of the La-Schiff base@MCM-41 catalyst, which indicates the successful stabilization of the lanthanum Schiff-base complex on the modified MCM-41. Furthermore, the EDS diagram of the La-Schiff base@MCM-41 catalyst shows the presence of chlorine element in its structure, which is related to some chlorine in 3-CPTMS@MCM-41 that was not replaced by the Schiff-base ligand 3. Also, the results of elemental composition of the La-Schiff base@MCM-41 catalyst from EDS analysis were confirmed by wavelength dispersive X-ray spectroscopy (WDX) analysis, which is shown in Fig. 3. The obtained results from WDX clearly show a homogeneous distribution of Si, O, C, N, Br, Cl and La in the structure of the La-Schiff base@MCM-41 catalyst. The results of WDX, like EDS, show that silicon and oxygen are the most common elements of the La-Schiff base@MCM-41 catalyst.

Considering that lanthanum metal is the active site of the La-Schiff base@MCM-41 catalyst, the exact amount of lanthanum element in La-Schiff base@MCM-41 was obtained by ICP analysis, and the value was 0.23 × 10^−3^ mmol g^−1^. Also, the content of carbon, hydrogen and nitrogen in La-Schiff base@MCM-41 was obtained using CHN analysis. The obtained results from CHN showed that this catalyst contains 9.17% carbon, 2.52% hydrogen and 0.22% nitrogen.

**Fig. 4:**
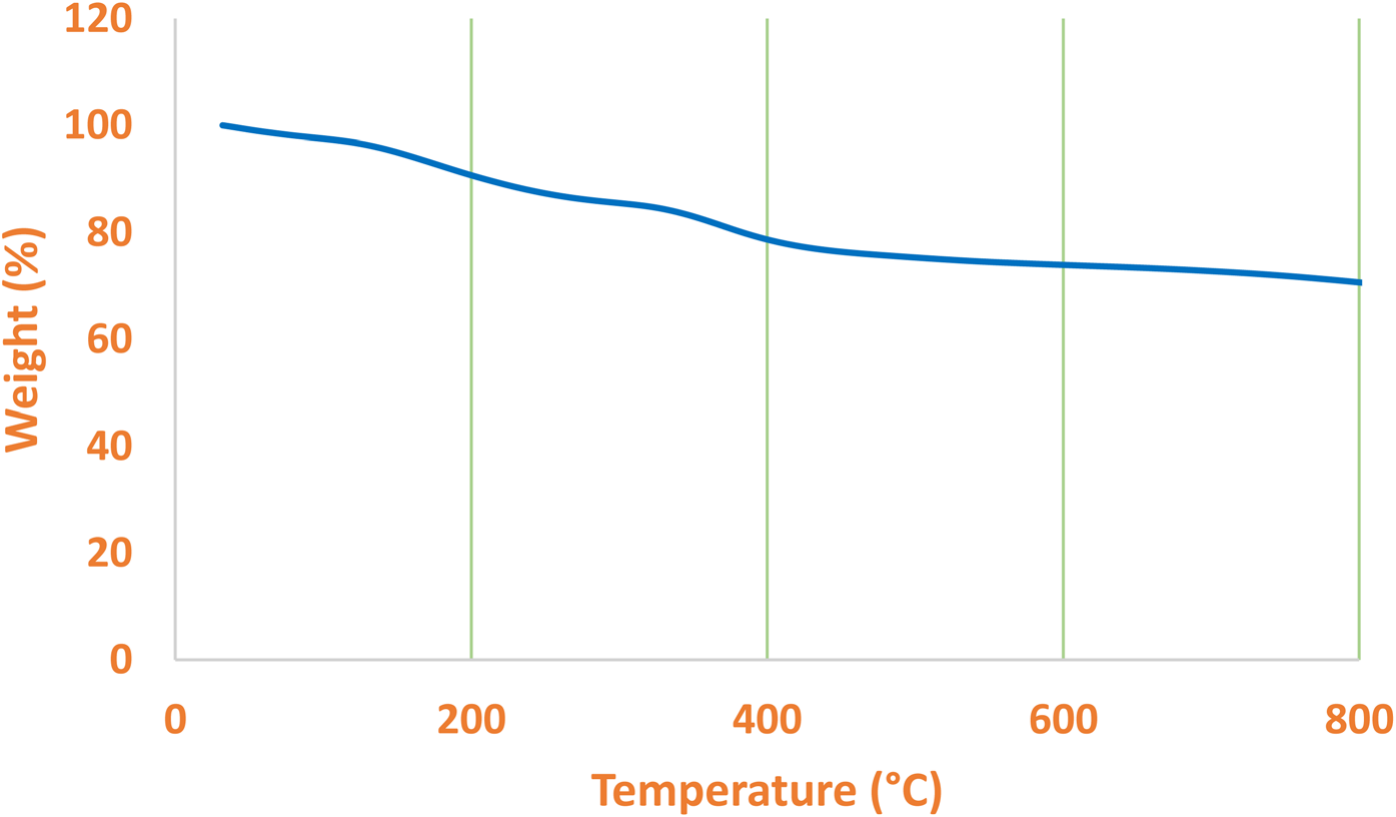
TGA diagram of La-Schiff base@MCM-41 catalyst.

Volatile components can be measured by mass loss in TGA. Therefore, TGA can be employed to determine the organic and inorganic component ratio in a sample. For this, TGA was performed by gradually raising the temperature of the La-Schiff base@MCM-41 catalyst (Fig. 4). The TGA diagram of the La-Schiff base@MCM-41 catalyst indicated a small mass loss (3% weight) below 150 °C, which is attributed to the evaporation of adsorbed solvents.^56^ Also, the TGA diagram of the La-Schiff base@MCM-41 catalyst indicated a notable mass loss (24% weight) in the region of 150 °C–600 °C, which represented the decomposition of immobilized Schiff-base organic components on the surface of MCM-41.^57^ Except for the evaporation of surface solvents, no weight loss was observed up to 160 °C, so this catalyst has thermal stability at least up to 160 °C.

**Fig. 5:**
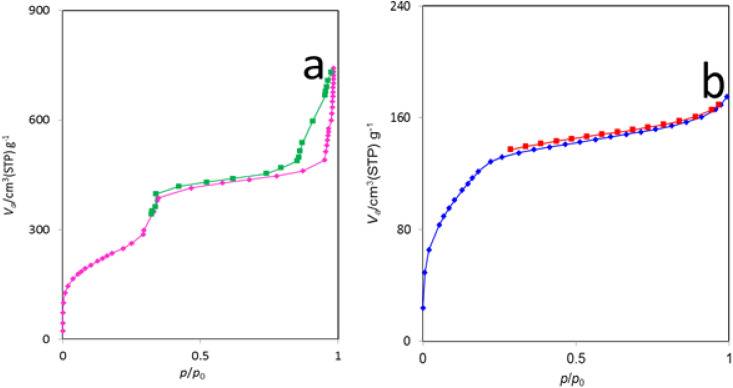
N_2_ adsorption–desorption isotherms of (a) MCM-41 and (b) La-Schiff base@MCM-41 catalyst.

The N_2_ adsorption–desorption isotherms of MCM-41 and the La-Schiff base@MCM-41 catalyst are shown in Fig. 5. Based on the IUPAC classification, this catalyst displays a type IV isotherm, which represents the class of mesoporous materials.^18,58^ The BET surface area of La-Schiff base@MCM-41 is 452.58 m^2^ g^−1^ (Table 1, entry 2). Due to its high surface area, it can be used as a catalyst. Also, the average pore diameter and pore volume of La-Schiff base@MCM-41 were calculated by the BET method using N_2_ adsorption–desorption and are 2.38 nm and 0.269 cm^3^ g^−1^, respectively (Table 1, entry 2). The BET surface area, average pore diameter and pore volume of MCM-41 are 854.13 m^2^ g^−1^, 5.37 nm and 1.15 cm^3^ g^−1^, respectively (Table 1, entry 1). The BET surface area, average pore diameter and pore volume of La-Schiff base@MCM-41 are lower than those of the unmodified mesoporous MCM-41 due to the grafting of the Schiff-base complex on MCM-41 (Table 1).^38,59^ The reason for the decrease in BET surface area, average pore diameter and pore volume is that when organic groups and metal complexes are stabilized inside the pores of MCM-41, the pores of MCM-41 are filled, therefore the volume of the pores, diameter of the pores and the surface area are also reduced.

**Table 1:** Textural properties of MCM-41 and La-Schiff base@MCM-41 catalyst

Entry	Sample	*S*_BET_ (m^2^ g^−1^)	Pore diameter (nm)	Pore volume (cm^3^ g^−1^)
1	MCM-41	854.13	5.37	1.15
2	La-Schiff base@MCM-41	452.58	2.381	0.269

**Fig. 6:**
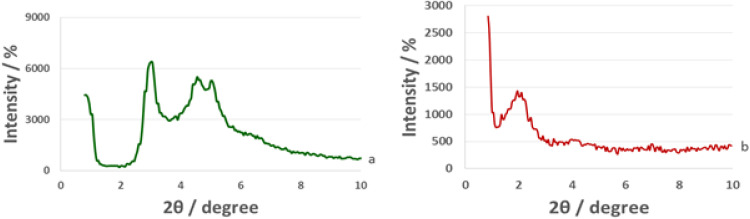
Low angle XRD patterns of (a) MCM-41 and (b) La-Schiff base@MCM-41 catalyst.

The small angle XRD patterns of MCM-41 and La-Schiff base@MCM-41 are shown in Fig. 6. The low angle XRD pattern of unfunctionalized MCM-41 has three peaks at 2*θ* values of about 2.95° (related to 100 reflections), 4.55° (related to 110 reflections) and 4.05° (related to 200 reflections), which represent the ordered hexagonal channels of MCM-41.^40^ As is well known, functionalization and modifying the MCM-41 surface can reduce or fade the intensity of these peaks. As predicted, these peaks are present in the low angle XRD pattern of La-Schiff base@MCM-41 with a lower intensity than those of the un-functionalized MCM-41, which is due to the grafting of the Schiff-base complex on MCM-41.^60^ The reason for the decrease in intensity of the peaks is that when organic groups and metal complexes are stabilized inside the pores of MCM-41, the ordered hexagonal nature of the channels is reduced.

### 3.2 Catalytic study of the catalyst

The catalytic ability of La-Schiff base@MCM-41 in organic chemistry was investigated in the synthesis of heterocyclic tetrazole compounds through the [3 + 2] cycloaddition of sodium azide (NaN_3_) with benzonitrile derivatives. For this study, the reaction of NaN_3_ with benzonitrile under various conditions was selected to find the best reaction conditions. In this regard, the effect of all parameters such as amount of NaN_3_, amount of La-Schiff base@MCM-41 catalyst, temperature and nature of solvent was examined in this reaction (Table 2). As shown in Table 2 (entry 2), 40 mg of La-Schiff base@MCM-41 catalyst cannot achieve completion of the reaction, while this reaction is completed when 50 mg of La-Schiff base@MCM-41 catalyst is used (Table 2, entry 3). Also, polar solvents provide better conditions for the synthesis of tetrazoles, therefore several polar solvents were examined to find the best reaction conditions for the [3 + 2] cycloaddition of NaN_3_ and benzonitrile (Table 2, entries 4–6). Also, the reaction was investigated in other solvents (such as dichloromethane and *n*-hexane), however the obtained results in these solvents were not favorable compared to the PEG-400 solvent (Table 2, entries 7 and 8). Because the boiling point of these solvents is low, the necessary temperature for the synthesis of tetrazole cannot be reached. Therefore, PEG-400 solvent was selected as the best solvent. Finally, the effect of temperature was examined (Table 2, entry 9), and the best results were obtained at 120 °C (Table 2, entry 3). The concentration of NaN_3_ was also checked, and 1.4 mmol of NaN_3_ was used for the synthesis of the tetrazole compounds.

**Table 2:** Study to find the best reaction conditions for the synthesis of 5-substituted 1*H*-tetrazoles in the presence of the La-Schiff base@MCM-41 nanocatalyst

Entry	Amount of catalyst (mg)	Solvent	NaN_3_ (mmol)	Time (min)	Temperature (°C)	Yield^a^ (%)
1	—	PEG	1.4	140	120	NR
2	40	PEG	1.4	270	120	90
3	50	PEG	1.4	120	120	98
4	50	PEG	1.3	150	120	75
5	50	DMSO	1.4	120	120	68
6	50	H_2_O	1.4	120	Reflux	55
7	50	*n*-Hexane	1.4	120	Reflux	Trace
8	50	Dichloromethane	1.4	120	Reflux	Trace
9	50	PEG	1.4	120	100	42

a Isolated yield.

**Scheme 4:**
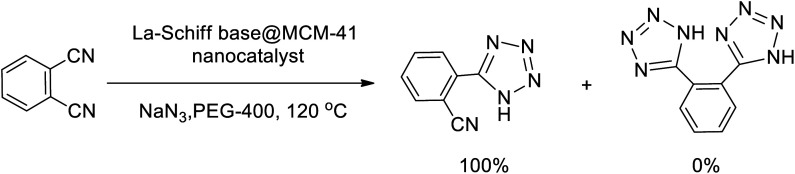
Homoselectivity of the La-Schiff base@MCM-41 nanocatalyst in the synthesis of 5-substituted 1*H*-tetrazoles from the [3 + 2] cycloaddition of NaN_3_ with dicyano substituted derivatives.

In continuation, several tetrazole compounds were synthesized under the optimized conditions in the presence of the La-Schiff base@MCM-41 catalyst (Table 3). Amongst these, various benzonitrile derivatives having electron-donating or electron-withdrawing functional groups were investigated. In these studies, all 5-substituted 1*H*-tetrazoles were synthesized in tolerable yields in the presence of the La-Schiff base@MCM-41 catalyst. As shown in Scheme 4, this catalyst shows excellent homoselectivity in the synthesis of 5-substituted 1*H*-tetrazoles. Further, in the reaction of NaN_3_ with phthalonitrile having the same two cyano-functional groups, only mono-addition was observed (Scheme 4).

**Table 3:** Synthesis of 5-substituted 1*H*-tetrazole derivatives catalyzed by the La-Schiff base@MCM-41 nanocatalyst

Entry	Nitrile	Product	Time (min)	Yield (%)	Melting point
1	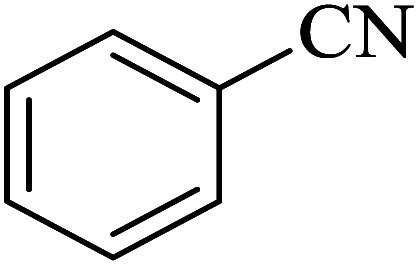	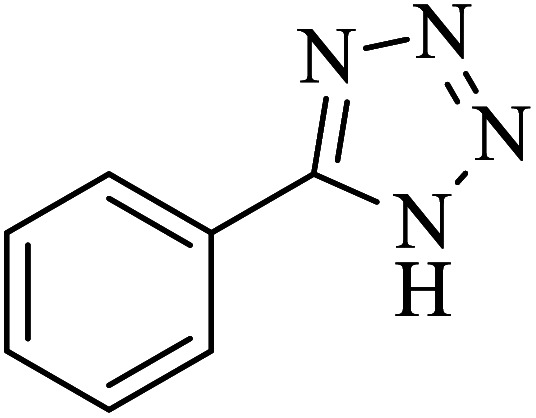	120	98	214–216
2	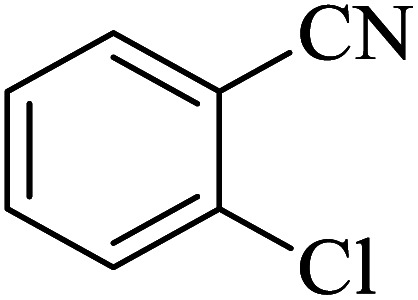	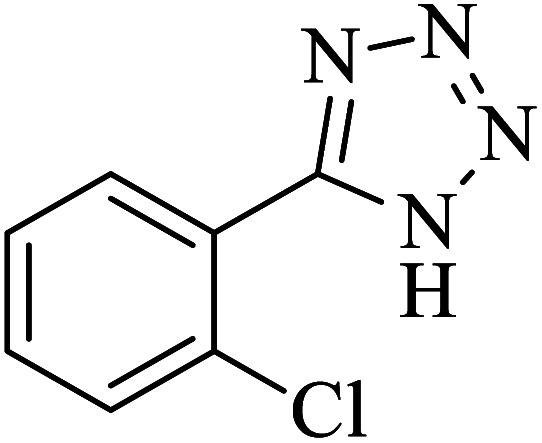	40	97	169–182
3	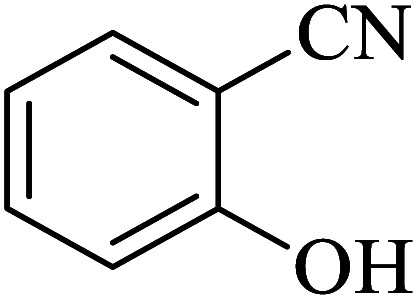	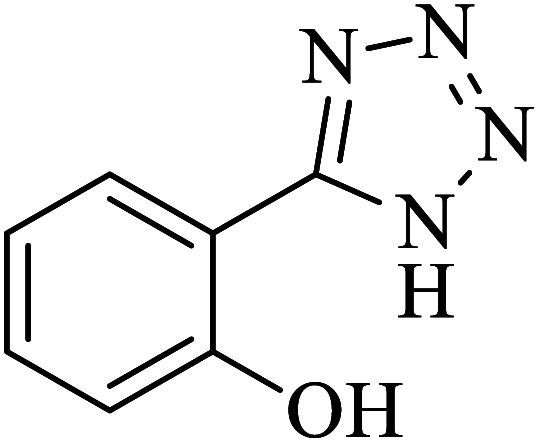	60	95	222–225
4	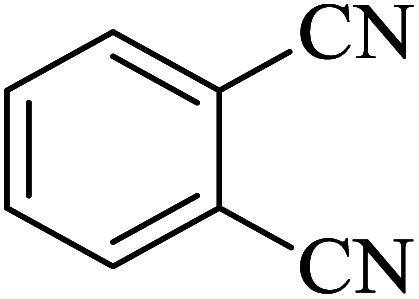	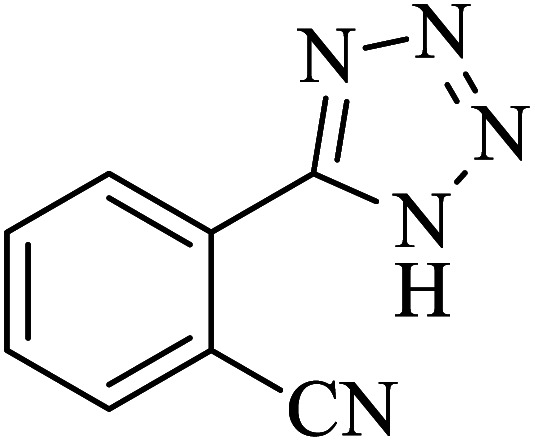	60	95	209–212
5	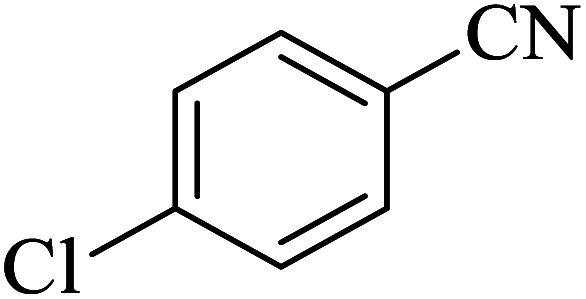	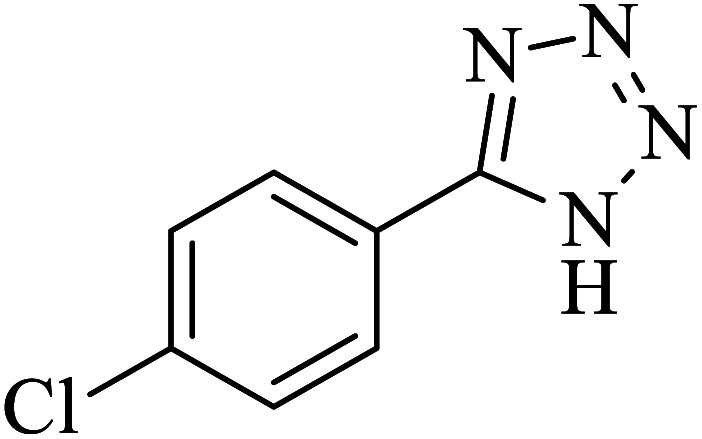	150	97	260–263
6	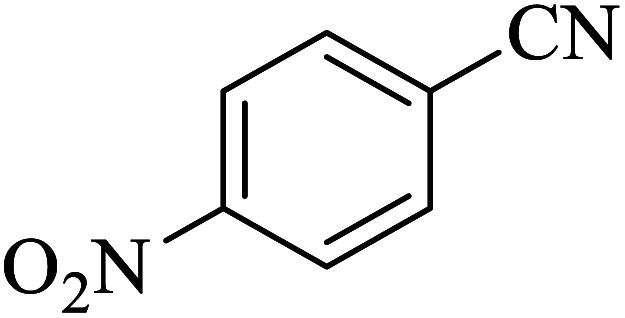	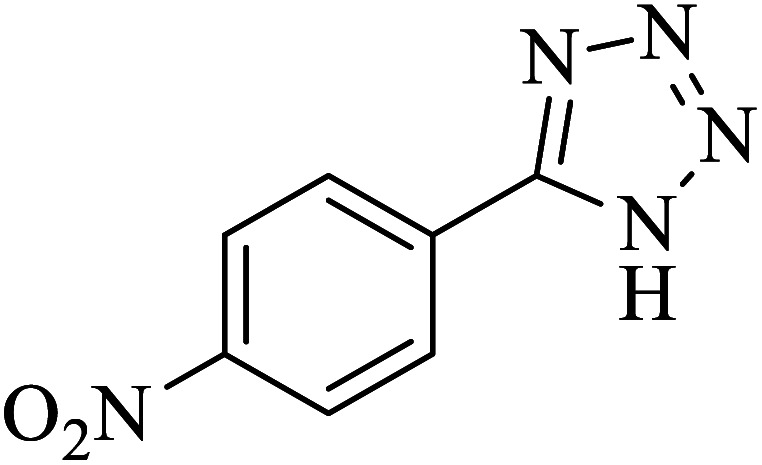	480	95	216–219
7	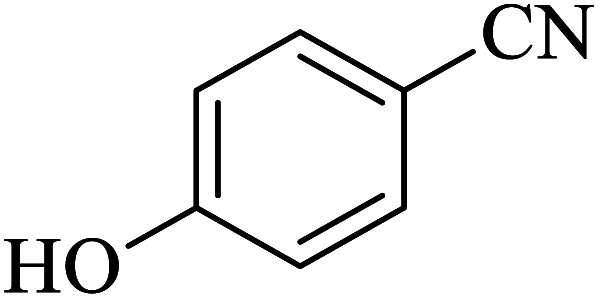	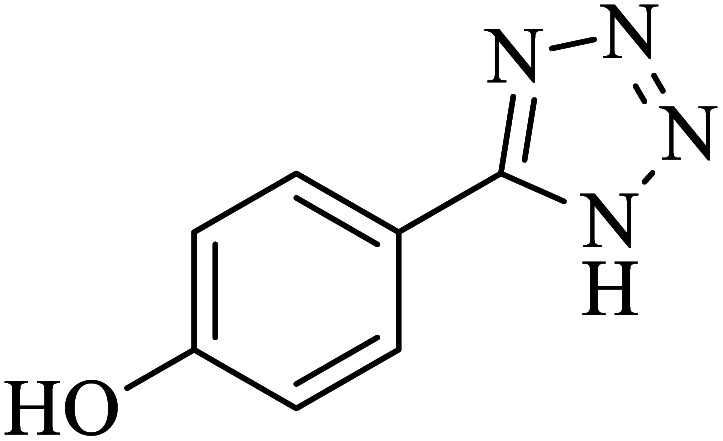	45	94	229–233
8	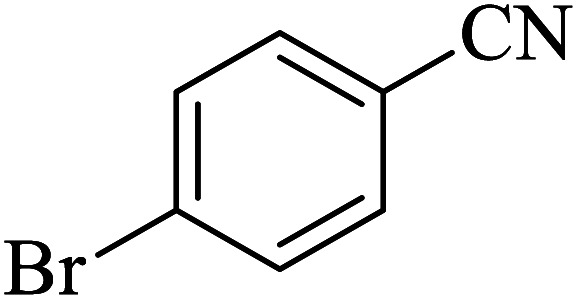	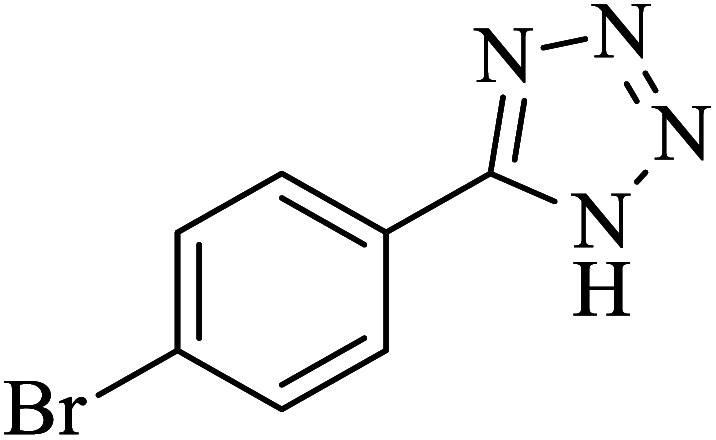	19h	89	259–262
9	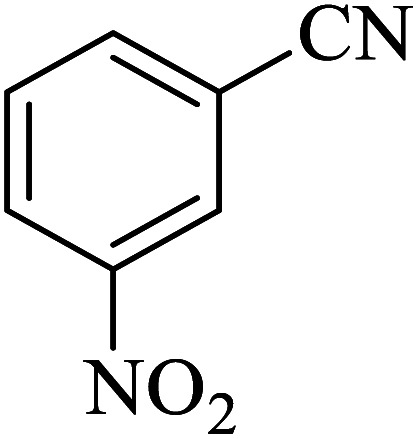	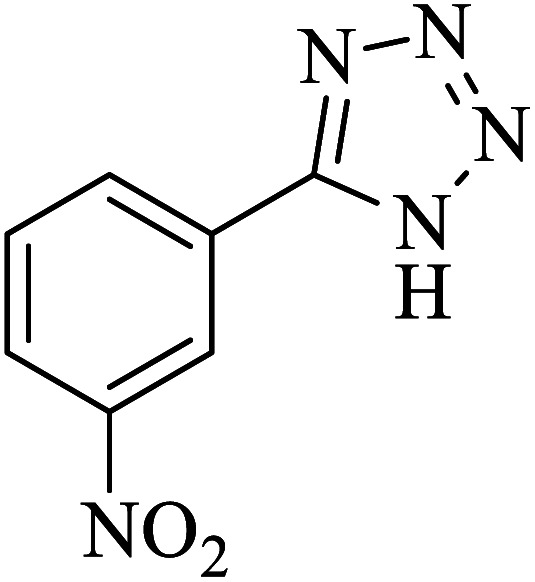	410	91	149–152

**Scheme 5:**
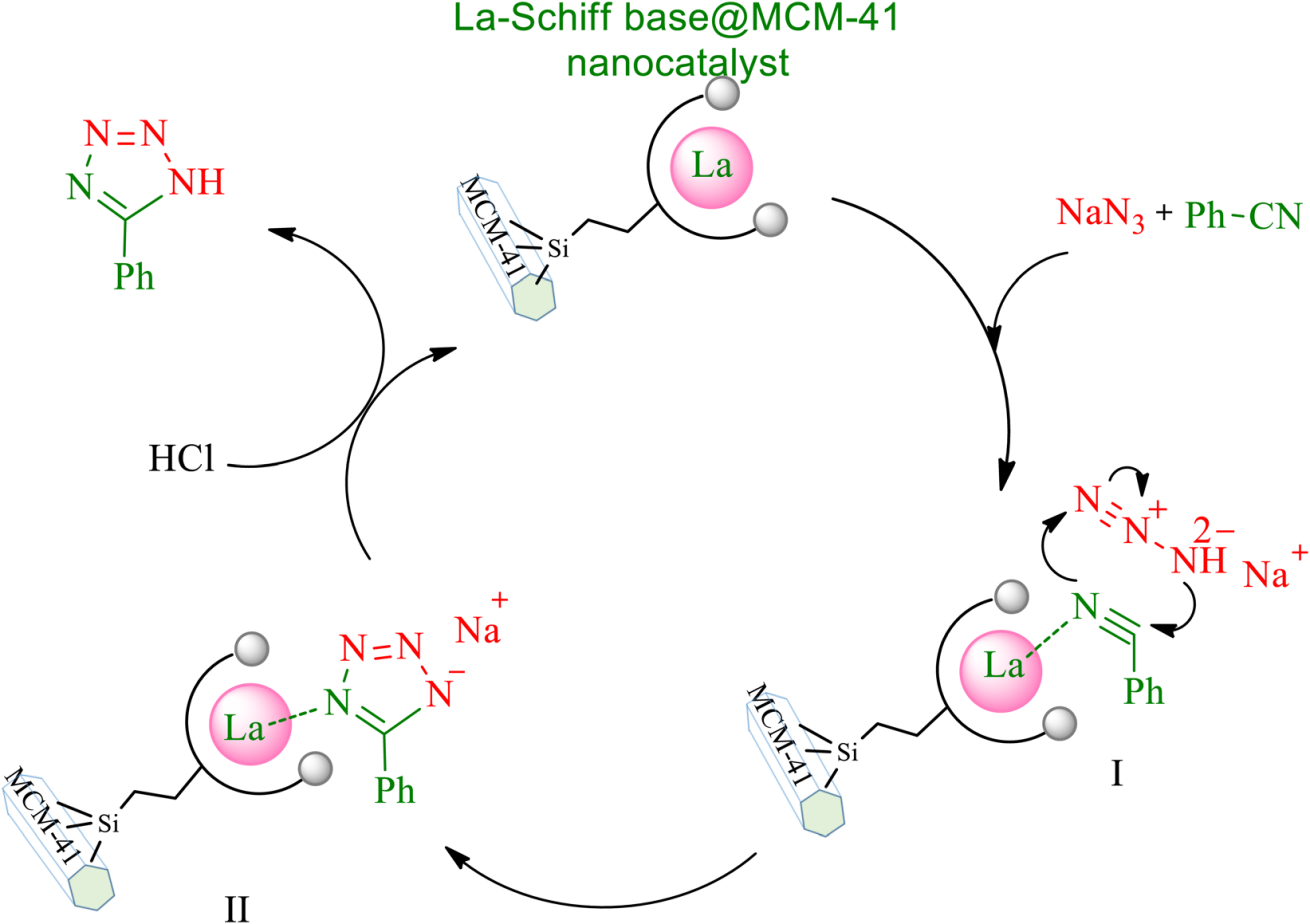
Expected mechanism for the synthesis of 5-substituted 1*H*-tetrazoles in the presence of the La-Schiff base@MCM-41 nanocatalyst.

Based on previous literature,^61,62^ a suitable cyclic mechanism for the synthesis of 5-substituted 1*H*-tetrazoles in the presence of the La-Schiff base@MCM-41 catalyst is proposed in Scheme 5. In this mechanism, intermediate I is formed from the interaction of the C≡N bond with the catalyst, which then introduced intermediate II. Then, in the work-up step, intermediate II gives the final tetrazole product by addition of HCl and the catalyst is regenerated and can undergo another catalytic cycle of the reaction.

### 3.3 Reusability of the catalyst

**Fig. 7:**
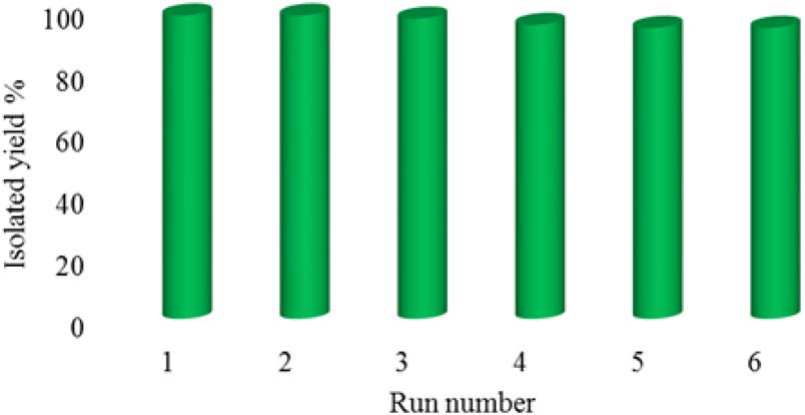
The recoverability and reusability of the La-Schiff base@MCM-41 nanocatalyst in the synthesis of 5-phenyl-1*H*-tetrazole.

The most important advantage of a heterogeneous catalyst over homogeneous catalysts is its recyclability. Accordingly, green chemistry recommends the use of heterogeneous catalysts because they are both environmentally friendly and economical. Therefore, the reusability of the La-Schiff base@MCM-41 catalyst was studied in the [3 + 2] cycloaddition of NaN_3_ with benzonitrile under the optimized conditions. In this regard, the catalyst was separated by centrifugation after each cycle and further was employed in the next cycle without any activation. As shown in Fig. 7, the La-Schiff base@MCM-41 catalyst can reused up to 6 times without a notable reduction of its catalytic efficiency.

### 3.4 Characterization of the recovered catalyst

The recovered La-Schiff base@MCM-41 catalyst was characterized using FT-IR spectroscopy, SEM and AAS.

To investigate the heterogeneous nature of the La-Schiff base@MCM-41 catalyst, the reaction between NaN_3_ and benzonitrile was performed under the optimized conditions. After completion of the reaction, the catalyst was removed by filtration. Then, the exact amount of leached lanthanum in the reaction solution was measured by atomic absorption spectroscopy (AAS). No significant amount of leached lanthanum was detected in the reaction solution. These results prove the heterogeneous nature and stability of the La-Schiff base@MCM-41 catalyst.

**Fig. 8:**
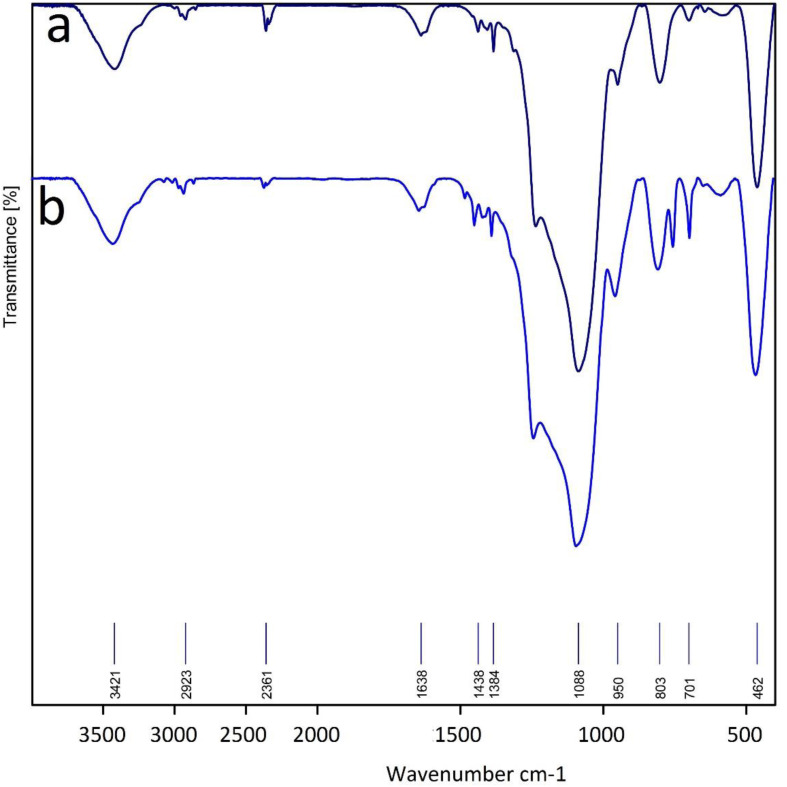
FT-IR spectra of (a) La-Schiff base@MCM-41 before reuse and (b) recovered La-Schiff base@MCM-41.

Also, the IR spectrum of the reused La-Schiff base@MCM-41 was compared to that of the fresh La-Schiff base@MCM-41 catalyst and a good similarity could be seen between them. The stability of La-Schiff base@MCM-41 was confirmed by the similar position and shape of the stretching vibrations in the FT-IR spectra of the recovered and fresh catalyst. These results provide strong evidence for the good stability of La-Schiff base@MCM-41 after reuse (Fig. 8).

In the IR spectra, three peaks at 462, 803, and 1088 cm^−1^ relate to the Si–O–Si vibrations.^63^ The stretching vibration of N–H and O–H bonds can be observed at 3417 cm^−1^.^10^ Also, stretching vibration of C–H bonds locates at 2923 cm^−1^ in the IR spectra.^21^ The bands at 701 cm^−1^, 1438 cm^−1^ and 1638 cm^−1^ correspond to the C–Br, aromatic C=C and C=N bonds, respectively.^10^

**Fig. 9:**
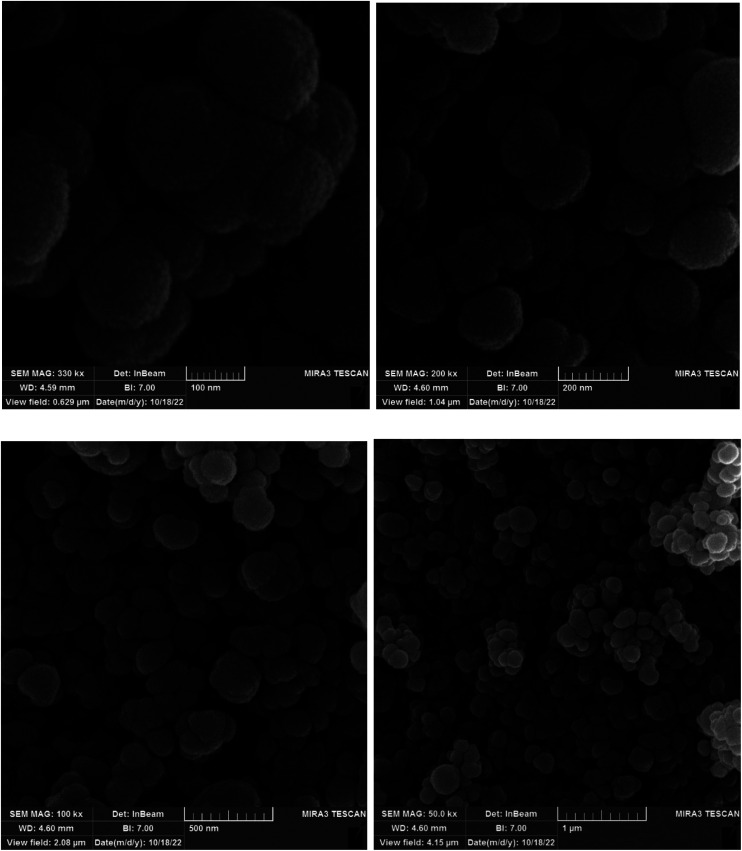
FESEM images of recovered La-Schiff base@MCM-41 catalyst.

The FESEM images of the recovered La-Schiff base@MCM-41 were obtained using an electron microscope (model MIRA3TESCAN-XMU). The FESEM images of the recovered La-Schiff base@MCM-41 are shown in Fig. 9. As shown, no significant change could be observed in the FESEM images of this catalyst after reuse in terms of shape or particle size. The SEM images of the recovered catalyst showed a good similarity with the SEM images of the fresh catalyst. Therefore, this catalyst is stable under the reaction conditions of the synthesis of tetrazoles.

### 3.5 Comparison of the catalyst

Table 4 is provided to show the practicality of the La-Schiff base@MCM-41 catalyst in comparison with some reported catalysts for the [3 + 2] cycloaddition of NaN_3_ with benzonitrile. As reported, the [3 + 2] cycloaddition of NaN_3_ with benzonitrile in the presence of unfunctionalized MCM-41 without any metal loading provided no 1*H*-tetrazole yield (Table 4, entry 1). However, the La-Schiff base@MCM-41 catalyst exhibited a 98% yield of 1*H*-tetrazole within 120 min, which is superior to some previously reported catalysts in terms of reaction times and yields. As shown in Table 4, the TON and TOF values of the La-Schiff base@MCM-41 catalyst are much higher than those of previous catalysts. TON and TOF are two important factors to evaluate the efficiency of catalysts. Therefore, one of the most important innovations of this work is the very high TON and TOF values of this La-Schiff base@MCM-41 catalyst compared to other catalysts. Previous methods using hazardous solvents and extremely long reaction times have been limited in the synthesis of 5-phenyl-1*H*-tetrazoles with biological properties. Now, using PEG as a green solvent, a short reaction time, excellent yield, easy recycling of the catalyst and pure separation of the products are the important advantages of the present strategy over previous strategies.

**Table 4:** Comparison results of the La-Schiff base@MCM-41 nanocatalyst with other catalysts in the synthesis of 5-phenyl-1*H*-tetrazole

Entry	Catalyst	Time (h)	Yield (%)	TON	TOF (h^−1^)	Ref.
1	MCM-41	2.5	Trace	—	—	18
2	CoY zeolite	14	90	—	—	64
3	Cu–Zn alloy nanopowder	10	95	—	—	65
4	B(C_6_F_5_)_3_	8	94	18.8	2.35	66
5	Fe_3_O_4_@SiO_2_/salen Cu(ii)	7	90	225	32.14	67
6	Fe_3_O_4_/ZnS HNSs	24	81.1	4.70	0.195	68
7	Pd-isatin-boehmite	8	94	21.3	2.6	69
8	Mesoporous ZnS	36	86	2.5	0.07	70
9	AgNO_3_	5	83	8300	1660	71
10	CuFe_2_O_4_	12	82	2.05	0.17	72
11	Nano ZnO/Co_3_O_4_	12	90	—	—	73
12	Pd-SMTU@boehmite	2.5	95	31.65	12.66	74
13	Cu-TBA@biochar	7	98	125.6	18.4	10
14	l-Cysteine-Pd@MCM-41	3	98	33.79	11.26	18
15	Ni-MP(AMP)_2_@Fe-biochar	3.8	92	1066	280.7	24
16	Cu(ii)-adenine-MCM-41	5	92	35.5	7.1	40
17	Pd-Arg@boehmite	7	97	28.7	4.1	57
18	Cu-DABP@Fe_3_O_4_/MCM-41	2	99	60	30	75
19	La-Schiff base@MCM-41	2	98	85 217	42 608	This work

## 4 Conclusions

In conclusion, we synthesized a new Schiff-base complex of lanthanum on MCM-41 (La-Schiff base@MCM-41). La-Schiff base@MCM-41 was characterized using various techniques such as ICP, CHN, XRD, TGA, BET, FT-IR spectroscopy, SEM, EDS and WDX. Then, the catalytic application of La-Schiff base@MCM-41 was studied in the homoselective synthesis of 5-substituted 1*H*-tetrazole derivatives. All products were obtained in good yields. Also, the recyclability of La-Schiff base@MCM-41 was described, which showed good recyclability in the synthesis of 5-substituted 1*H*-tetrazoles.

## Conflicts of interest

There are no conflicts to declare.

## Supplementary Material

RA-012-D2RA05413B-s001

## References

[cit1] Gupta P., Paul S. (2014). Solid acids: Green alternatives for acid catalysis. Catal. Today.

[cit2] Moradi P., Hajjami M. (2022). Stabilization of ruthenium on biochar-nickel magnetic nanoparticles as a heterogeneous, practical, selective, and reusable nanocatalyst for the Suzuki C–C coupling reaction in water. RSC Adv..

[cit3] Woo Lim C., Lee I. S. (2010). Magnetically recyclable nanocatalyst systems for the organic reactions. Nano Today.

[cit4] Gómez-López P., Puente-Santiago A., Castro-Beltrán A., Nascimento L. A. S., Balu A. M., Luque R., Alvarado-Beltrán C. G. (2020). Nanomaterials and catalysis for green chemistry. Curr. Opin. Green Sustainable Chem..

[cit5] Astruc D., Lu F., Aranzaes J. R. (2005). Nanoparticles as Recyclable Catalysts: The Frontier between Homogeneous and Heterogeneous Catalysis. Angew. Chem., Int. Ed..

[cit6] Polshettiwar V., Varma R. S. (2010). Green chemistry by nano-catalysis. Green Chem..

[cit7] Wang D., Astruc D. (2014). Fast-Growing Field of Magnetically Recyclable Nanocatalysts. Chem. Rev..

[cit8] Polshettiwar V., Luque R., Fihri A., Zhu H., Bouhrara M., Basset J. M. (2011). Magnetically Recoverable Nanocatalysts. Chem. Rev..

[cit9] Zhu Y., Stubbs L. P., Ho F., Liu R., Ship C. P., Maguire J. A., Hosmane N. S. (2010). Magnetic Nanocomposites: A New Perspective in Catalysis. ChemCatChem.

[cit10] Moradi P., Hajjami M., Tahmasbi B. (2020). Fabricated copper catalyst on biochar nanoparticles for the synthesis of tetrazoles as antimicrobial agents. Polyhedron.

[cit11] Huang W., Jiang J., Sanchez-Mendoza A. (2021). Synthesis of heterocycles catalyzed by mesoporous silica MCM nanoparticles. Synth. Commun..

[cit12] Biradha K., Goswami A., Moi R. (2020). Coordination polymers as heterogeneous catalysts in hydrogen evolution and oxygen evolution reactions. Chem. Commun..

[cit13] Corcho-Valdés A. L., Iriarte-Mesa C., Calzadilla-Maya J., Matos-Peralta Y., Desdín-García L. F., Antuch M. (2022). Carbon Nanotubes in Organic Catalysis. Carbon Composite Catalysts.

[cit14] Melchionna M., Marchesan S., Prato M., Fornasiero P. (2015). Carbon nanotubes and catalysis: the many facets of a successful marriage. Catal. Sci. Technol..

[cit15] Li T. T., Mei Y., Li H., Qian J., Wu M., Zheng Y. Q. (2020). Highly Selective and Active Electrochemical Reduction of CO_2_ to CO on a Polymeric Co(II) Phthalocyanine@Graphitic Carbon Nitride Nanosheet−Carbon Nanotube Composite. Inorg. Chem..

[cit16] Nikoorazm M., Rezaei Z., Tahmasbi B. (2020). Two Schiff-base complexes of copper and zirconium oxide supported on mesoporous MCM-41 as an organic–inorganic hybrid catalysts in the chemo and homoselective oxidation of sulfides and synthesis of tetrazoles. J. Porous Mater..

[cit17] Eslami M., Dekamin M. G., Motlagh L., Maleki A. (2018). MCM-41 mesoporous silica: a highly efficient and recoverable catalyst for rapid synthesis of α-aminonitriles and imines. Green Chem. Lett. Rev..

[cit18] Nikoorazm M., Moradi P., Noori N. (2020). L-cysteine complex of palladium onto mesoporous channels of MCM-41 as reusable, homoselective and organic–inorganic hybrid nanocatalyst for the synthesis of tetrazoles. J. Porous Mater..

[cit19] Ghorbani-Choghamarani A., Moradi P., Tahmasbi B. (2019). Modification of boehmite nanoparticles with Adenine for the immobilization of Cu(II) as organic–inorganic hybrid nanocatalyst in organic reactions. Polyhedron.

[cit20] Baran N. Y., Baran T., Nasrollahzadeh M., Varma R. S. (2019). Pd nanoparticles stabilized on the Schiff base-modified boehmite: Catalytic role in Suzuki coupling reaction and reduction of nitroarenes. J. Organomet. Chem..

[cit21] Jabbari A., Moradi P., Hajjami M., Tahmasbi B. (2022). Tetradentate copper complex supported on boehmite nanoparticles as an efficient and heterogeneous reusable nanocatalyst for the synthesis of diaryl ethers. Sci. Rep..

[cit22] Ghorbani-Choghamarani A., Moradi P., Tahmasbi B. (2019). Nickel(II) immobilized on dithizone–boehmite nanoparticles: as a highly efficient and recyclable nanocatalyst for the synthesis of polyhydroquinolines and sulfoxidation reaction. J. Iran. Chem. Soc..

[cit23] Moradi P., Hajjami M. (2021). Magnetization of graphene oxide nanosheets using nickel magnetic nanoparticles as a novel support for the fabrication of copper as a practical, selective, and reusable nanocatalyst in C–C and C–O coupling reactions. RSC Adv..

[cit24] Moradi P., Hajjami M. (2021). Magnetization of biochar nanoparticles as a novel support for fabrication of organo nickel as a selective, reusable and magnetic nanocatalyst in organic reactions. New J. Chem..

[cit25] Moradi P., Hajjami M., Valizadeh-Kakhki F. (2019). Biochar as heterogeneous support for immobilization of Pd as efficient and reusable biocatalyst in C–C coupling reactions. J. Organomet. Chem..

[cit26] Ghorbani-Choghamarani A., Tahmasbi B., Moradi Z. (2017). S- Benzylisothiourea complex of palladium on magnetic nanoparticles: A highly efficient and reusable nanocatalyst for synthesis of polyhydroquinolines and Suzuki reaction. J. Organomet. Chem..

[cit27] Ghorbani-Choghamarani A., Rabiei H., Tahmasbi B., Ghasemi B., Mardi F. (2016). Preparation of DSA@MNPs and application as heterogeneous and recyclable nanocatalyst for oxidation of sulfides and oxidative coupling of thiols. Res. Chem. Intermed..

[cit28] Rezaei A., Ghorbani-Choghamarani A., Tahmasbi B. (2022). Synthesis and Characterization of Nickel Metal-Organic Framework Including 4,6-diamino-2-mercaptopyrimidine and its Catalytic Application in Organic Reactions. Catal. Lett..

[cit29] Wu Y. L., Li X., Wei Y. S., Fu Z., Wei W., Wu X. T., Zhu Q. L., Xu Q. (2021). Ordered Macroporous Superstructure of Nitrogen-Doped Nanoporous Carbon Implanted with Ultrafine Ru Nanoclusters for Efficient pH-Universal Hydrogen Evolution Reaction. Adv. Mater..

[cit30] Mei Y., Li T. T., Qian J., Li H., Wu M., Zheng Y. Q. (2020). Construction of C@MoS_2_@C Sandwiched Heterostructure for Accelerating pH-Universal Hydrogen Evolution Reaction. Chem. Commun..

[cit31] Vallet-Regi M., Balas F., Arcos D. (2007). Mesoporous materials for drug delivery. Angew. Chem., Int. Ed..

[cit32] Galhano J., Marcelo G. A., Duarte M. P., Oliveira E. (2022). Ofloxacin@Doxorubicin-Epirubicin functionalized MCM-41 mesoporous silica–based nanocarriers as synergistic drug delivery tools for cancer related bacterial infections. Bioorg. Chem..

[cit33] Kumar S., Bhogal S., Sharma P., Rani S., Aulakh J. S., Malik A. K. (2022). Mobil catalytic material number 41 modified magnetite nano-composites for efficient extraction of non-steroidal anti-inflammatory drugs from tap water and urine samples. Sep. Sci. plus.

[cit34] Ma M., Gao K., Zhao D., Ma X., Ma Z. (2022). Effect of process conditions on reaction-type adsorption of o-xylene by MCM-41 supported sulfuric acid: Model simulations of breakthrough curves. J. Environ. Chem. Eng..

[cit35] Zhang L., Qi L., Han Y., Fei Z., Chen X., Zhang Z., Tang J., Cui M., Qiao X., Liu Q. (2022). Amino-Functionalized Pore-Expanded MCM-41 for CO2 Adsorption: Effect of Alkyl Chain Length of the Template. Ind. Eng. Chem. Res..

[cit36] Ghalkhani M., Sohouli E. (2022). Synthesis of the decorated carbon nano onions with aminated MCM-41/Fe3O4 NPs: Morphology and electrochemical sensing performance for methotrexate analysis. Microporous
Mesoporous Mater..

[cit37] Faramarzi M., Khanmohammadi H., Zendehdel M. (2022). Novel MCM-41 based chromogenic probe: highly selective and sensitive upon cyanide ion over other interfering anions in water. J. Porous Mater..

[cit38] Nikoorazm M., Tahmasbi B., Gholami S., Moradi P. (2020). Copper and nickel immobilized on cytosine@MCM-41: as highly efficient, reusable and organic–inorganic hybrid nanocatalysts for the homoselective synthesis of tetrazoles and pyranopyrazoles. Appl. Organomet. Chem..

[cit39] Nikoorazm M., Noori N., Tahmasbi B., Faryadi S. (2017). A palladium complex immobilized onto mesoporous silica: a highly efficient and reusable catalytic system for carbon–carbon bond formation and anilines synthesis. Transition Met. Chem..

[cit40] Nikoorazm M., Ghorbani-Choghamaranai A., Khanmoradi M., Moradi P. (2018). Synthesis and characterization of Cu(II)-Adenine-MCM-41 as stable and efficient mesoporous catalyst for the synthesis of 5-substituted 1H-tetrazoles and 1H-indazolo [1,2-b]phthalazine-triones. J. Porous Mater..

[cit41] Sahoo D. P., Rath D., Nanda B., Parida K. M. (2015). Transition metal/metal oxide modified MCM-41 for pollutant degradation and hydrogen energy production: a review. RSC Adv..

[cit42] Liu C. (2021). Based on MCM nanomaterials: Recoverable metallic nanocatalysts in oxidation of sulfides and oxidative coupling of thiols. Synth. Commun..

[cit43] Costa J. A. S., Jesus R. A., Santos D. O., Mano J. F., Romão L. P. C., Paranhos C. M. (2020). Recent progresses in the adsorption of organic, inorganic, and gas compounds by MCM-41-based mesoporous materials. Microporous Mesoporous Mater..

[cit44] Karger-Kocsis J., Lendvai L. (2018). Polymer/boehmite nanocomposites: A review. J. Appl. Polym. Sci..

[cit45] Neochoritis C. G., Zhao T., Dömling A. (2019). Tetrazoles via multicomponent reactions. Chem. Rev..

[cit46] Hamrahian S. A., Salehzadeh S., Rakhtshah J., Haji babaei F., Karami N. (2019). Preparation, characterization and catalytic application of molybdenum Schiff-base complex immobilized on silica-coated Fe_3_O_4_ as a reusable catalyst for the synthesis of pyranopyrazole derivatives. Appl. Organomet. Chem..

[cit47] Samanta P. K., Biswas R., Das T., Nandi M., Adhikary B., Richards R. M., Biswas P. (2019). Mesoporous silica supported samarium as recyclable heterogeneous catalyst for synthesis of 5-substituted tetrazole and 2-substituted benzothiazole. J. Porous Mater..

[cit48] Rezaei F., Ali Amrollahi M., Khalifeh R. (2019). Design and synthesis of Fe3O4@SiO2/aza-crown ether-Cu(II) as a novel and highly efficient magnetic nanocomposite catalyst for the synthesis of 1,2,3-triazoles, 1-substituted 1H-tetrazoles and 5-substituted 1H-tetrazoles in green solvents. Inorg. Chim. Acta.

[cit49] Akbarzadeh P., Koukabi N., Kolvari E. (2019). Three-component solvent-free synthesis of 5-substituted-1H-tetrazoles catalyzed by unmodified nanomagnetite with microwave irradiation or conventional heating. Res. Chem. Intermed..

[cit50] Maleki A., Niksefat M., Rahimi J., Azadegan S. (2019). Facile synthesis of tetrazolo [1, 5-a] pyrimidine with the aid of an effective gallic acid nanomagnetic catalyst. Polyhedron.

[cit51] Sarvary A., Maleki A. (2015). A review of syntheses of 1, 5-disubstituted tetrazole derivatives. Mol. Diversity.

[cit52] Maleki A., Sarvary A. (2015). Synthesis of tetrazoles via isocyanide-based reactions. RSC Adv..

[cit53] Kant R., Singh V., Agarwal A. (2016). An efficient and economical synthesis of 5-substituted 1H-tetrazoles via Pb (II) salt catalyzed [3+ 2] cycloaddition of nitriles and sodium azide. C. R. Chim..

[cit54] Kumar Samanta P., Biswas R., Das T., Nandi M., Adhikary B., Richards R. M., Biswas P. (2019). Mesoporous silica supported samarium as recyclable heterogeneous catalyst for synthesis of 5-substituted tetrazole and 2-substituted benzothiazole. J. Porous Mater..

[cit55] Ojeda-Carralero G. M., Coro J., Valdés-Palacios A. (2020). Green alternatives for the synthesis of tetrazolic acids. Chem. Heterocycl. Compd..

[cit56] Nikoorazm M., Moradi P., Noori N., Azadi G. (2021). L-Arginine complex of copper on modified core–shell magnetic nanoparticles as reusable and organic–inorganic hybrid nanocatalyst for the chemoselective oxidation of organosulfur compounds. J. Iran. Chem. Soc..

[cit57] Tahmasbi B., Ghorbani-Choghamarani A. (2017). First report of the direct supporting of palladium–arginine complex on boehmite nanoparticles and application in the synthesis of 5-substituted tetrazoles. Appl. Organomet. Chem..

[cit58] Muttakin M., Mitra S., Thu K., Ito K., Baran Saha B. (2018). Theoretical framework to evaluate minimum desorption temperature for IUPAC classified adsorption isotherms. Int. J. Heat Mass Transfer.

[cit59] Ghorbani-Choghamarani A., Nikpour F., Ghorbani F., Havasi F. (2015). Anchoring of Pd(II) complex in functionalized MCM-41 as an efficient and recoverable novel nanocatalyst in C–C, C–O and C–N coupling reactions using Ph3SnCl. RSC Adv..

[cit60] Viswanadham B., Vishwanathan V., Chary K. V. R., Satyanarayana Y. (2021). Catalytic dehydration of glycerol to acrolein over mesoporous MCM-41 supported heteropolyacid catalysts. J. Porous Mater..

[cit61] Tahmasbi B., Ghorbani-Choghamarani A., Moradi P. (2020). Palladium fabricated on boehmite as an organic–inorganic hybrid nanocatalyst for C–C cross coupling and homoselective cycloaddition reactions. New J. Chem..

[cit62] Abrishami F., Ebrahimikia M., Rafiee F. (2015). Synthesis of 5-substituted 1H-tetrazoles using a recyclable heterogeneous nanonickel ferrite catalyst. Appl. Organomet. Chem..

[cit63] Nikoorazm M., Ghorbani-Choghamarani A., Panahi A., Tahmasbi B., Noori N. (2018). Pd(0)-Schif-base@MCM-41 as high-efficient and reusable catalyst for C–C coupling reactions. J. Iran. Chem. Soc..

[cit64] Rama V., Kanagaraj K., Pitchumani K. (2011). Syntheses of 5-Substituted 1H-Tetrazoles Catalyzed by Reusable CoY Zeolite. J. Org. Chem..

[cit65] Aridoss G., Laali K. K. (2011). Highly Efficient Synthesis of 5-Substituted 1H -Tetrazoles Catalyzed by Cu–Zn Alloy Nanopowder, Conversion into 1,5- and 2,5-Disubstituted Tetrazoles, and Synthesis and NMR Studies of New Tetrazolium Ionic Liquids. Eur. J. Org. Chem..

[cit66] Kumar Prajapti S., Nagarsenkar A., Nagendra Babu B. (2014). An efficient synthesis of 5-substituted 1H-tetrazoles via B(C_6_F_5_)_3_ catalyzed [3+2] cycloaddition of nitriles and sodium azide. Tetrahedron Lett..

[cit67] Dehghani F., Sardarian A. R., Esmaeilpour M. (2013). Salen complex of Cu(II) supported on superparamagnetic Fe_3_O_4_@SiO_2_ nanoparticles: An efficient and recyclable catalyst for synthesis of 1- and 5-substituted 1H-tetrazoles. J. Organomet. Chem..

[cit68] Qi G., Liu W., Bei Z. (2011). Fe_3_O_4_/ZnS Hollow Nanospheres: A Highly Efficient Magnetic Heterogeneous Catalyst for Synthesis of 5-Substituted 1H -Tetrazoles from Nitriles and Sodium Azide. Chin. J. Chem..

[cit69] Jabbari A., Tahmasbi B., Nikoorazm M., Ghorbani-Choghamarani A. (2018). A new Pd-Schiff-base complex on boehmite nanoparticles: Its application in Suzuki reaction and synthesis of tetrazoles. Appl. Organomet. Chem..

[cit70] Lang L., Zhou H., Xue M., Wang X., Xu Z. (2013). Mesoporous ZnS hollow spheres-catalyzed synthesis of 5-substituted 1H-tetrazoles. Mater. Lett..

[cit71] Mani P., Singh A. K., Awasthi S. K. (2014). AgNO_3_ catalyzed synthesis of 5-substituted-1H-tetrazole via [3+2] cycloaddition of nitriles and sodium azide. Tetrahedron Lett..

[cit72] Sreedhar B., Suresh Kumar A., Yada D. (2011). CuFe_2_O_4_ nanoparticles: a magnetically recoverable and reusable catalyst for the synthesis of 5-substituted 1H-tetrazoles. Tetrahedron Lett..

[cit73] Agawane S. M., Nagarkar J. M. (2012). Synthesis of 5-substituted 1H-tetrazoles
using a nano ZnO/Co_3_O_4_ catalyst. Catal. Sci. Technol..

[cit74] Moradi P., Ghorbani-Choghamarani A. (2017). Efficient synthesis of 5-substituted tetrazoles catalysed by palladium–S-methylisothiourea complex supported on boehmite nanoparticles. Appl. Organomet. Chem..

[cit75] Kikhavani T., Moradi P., Mashari-Karir M., Naji J. (2022). A new copper Schiff-base complex of 3,4-diaminobenzophenone stabilized on magnetic MCM-41 as a homoselective and reusable catalyst in the synthesis of tetrazoles and pyranopyrazoles. Appl. Organomet. Chem..

