# Supplementary material for: A Schiff base complex of lanthanum on modified MCM-41 as a reusable nanocatalyst in the homoselective synthesis of 5-substituted 1H-tetrazoles
Source: RSC Adv. 2022 Nov 29;12(53):34303–17. doi: 10.1039/d2ra05413b (PMC9707292; doi:10.1039/d2ra05413b)
Supplement: RA-012-D2RA05413B-s001 [file RA-012-D2RA05413B-s001.pdf]

Electronic Supplementary Material (ESI) for RSC Advances.
This journal is © The Royal Society of Chemistry 2022

Supplementary Data

A Schiff base complex of lanthanum on modified MCM-41 as a reusable nanocatalyst in the homoselective synthesis of 5-substituted 1H-tetrazole derivatives

Bahman Tahmasbi*, Mohsen Nikoorazm*, Parisa Moradi, Yunes Abbasi Tyula

[b]*Department of Chemistry, Faculty of Science, Ilam University, P.O. Box 69315516, Ilam, Iran.*

Abstract:

In this work mesoporous MCM-41 was modified by a new Schiff-base from condensation of triethylenetatramine and 5-bromosalicylaldehyde. Then it was used for stabilization of lanthanum metal (La-Schiff base@MCM-41) as a homoselective, reusable, efficient and biocompatible in the synthesis of 5-substituted 1H-tetrazole derivatives which synthesized tetrazoles were characterized by ^{1}H NMR and FT-IR spectroscopy and physical properties. La-Schiff base@MCM-41 was characterized by various techniques such as ICP, CHN, XRD, TGA, BET, FT-IR, SEM, EDS and WDX. This catalyst has good stability and heterogeneity nature which can easily recovered and reused for several times without significant loss in catalytic activity. This present strategy has important advantages such as utilize PEG as green solvent, short reaction time, excellent yield, easy recycling of the catalyst and pure separation of the products. The recovered La-Schiff base@MCM-41 catalyst was characterized by FT-IR, SEM and AAS techniques.

Keywords: Mesoporous MCM-41; Heterogeneous catalyst; Schiff-base complex, Lanthanum, 5-substituted 1H-tetrazole.

---

* Address correspondence to Department of Chemistry, Faculty of Science, Ilam University, P. O. Box 69315516, Ilam, Iran. E-mail address of B. Tahmasbi: b.tahmasbi@ilam.ac.ir, bah.tahmasbi@gmail.com, and M. Nikoorazm: m.nikorazm@ilam.ac.ir, e_nikoorazm@yahoo.com

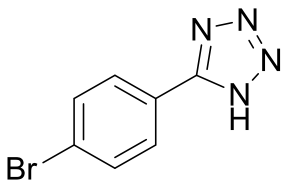

5-(4-bromophenyl)-1H-tetrazole: ^{1}H NMR (400 MHz, DMSO): δ_H= 7.98-7.95 (d, *J*= 12 Hz, 2H), 7.83-7.81 (d, *J*= 12 Hz, 2H) ppm.

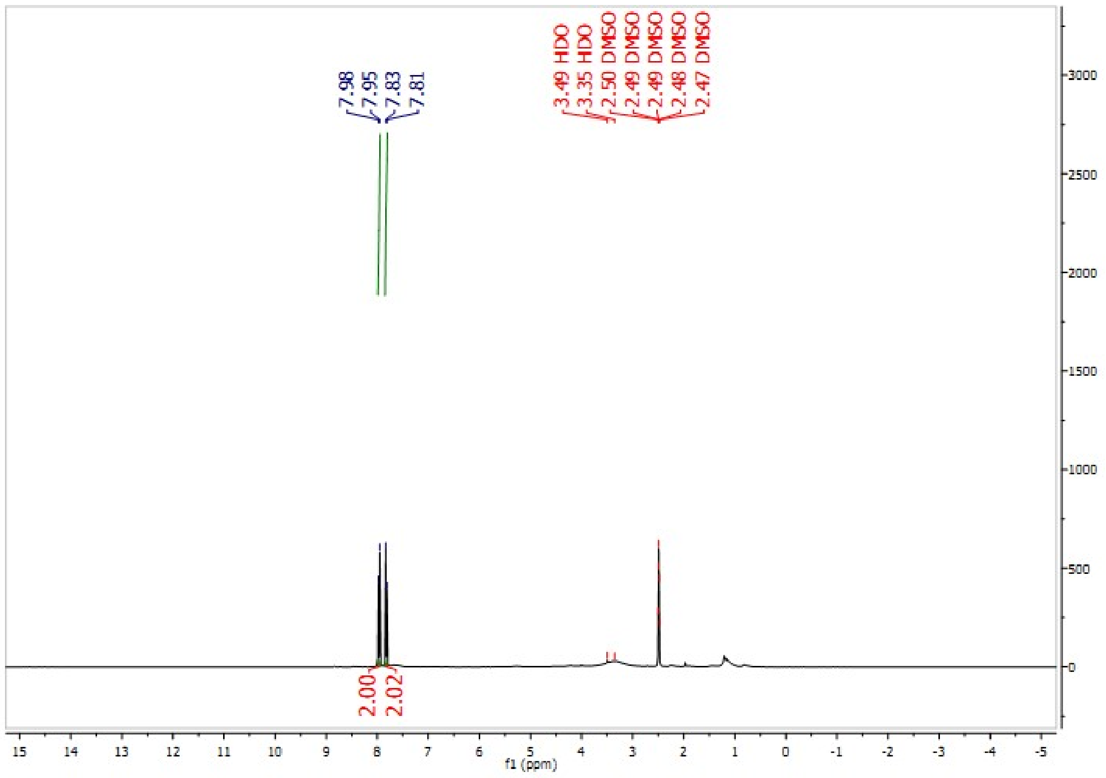

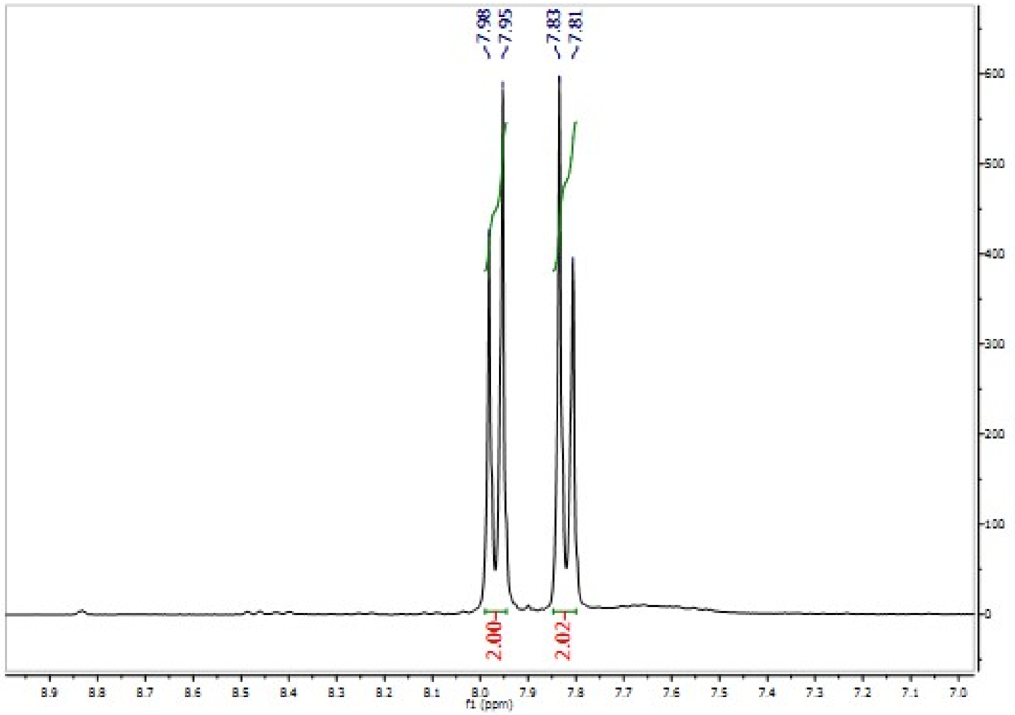

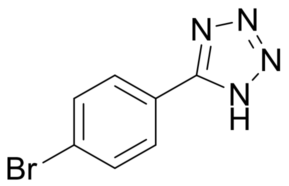

5-(4-bromophenyl)-1H-tetrazole: IR (KBr) cm^{-1}: 3423, 3090, 3064, 2966, 2852, 2768, 1638, 1611, 1559, 1482, 1431, 1385, 1276, 1252, 1157, 1119, 1054, 1018, 988, 872, 828, 742, 614, 451.

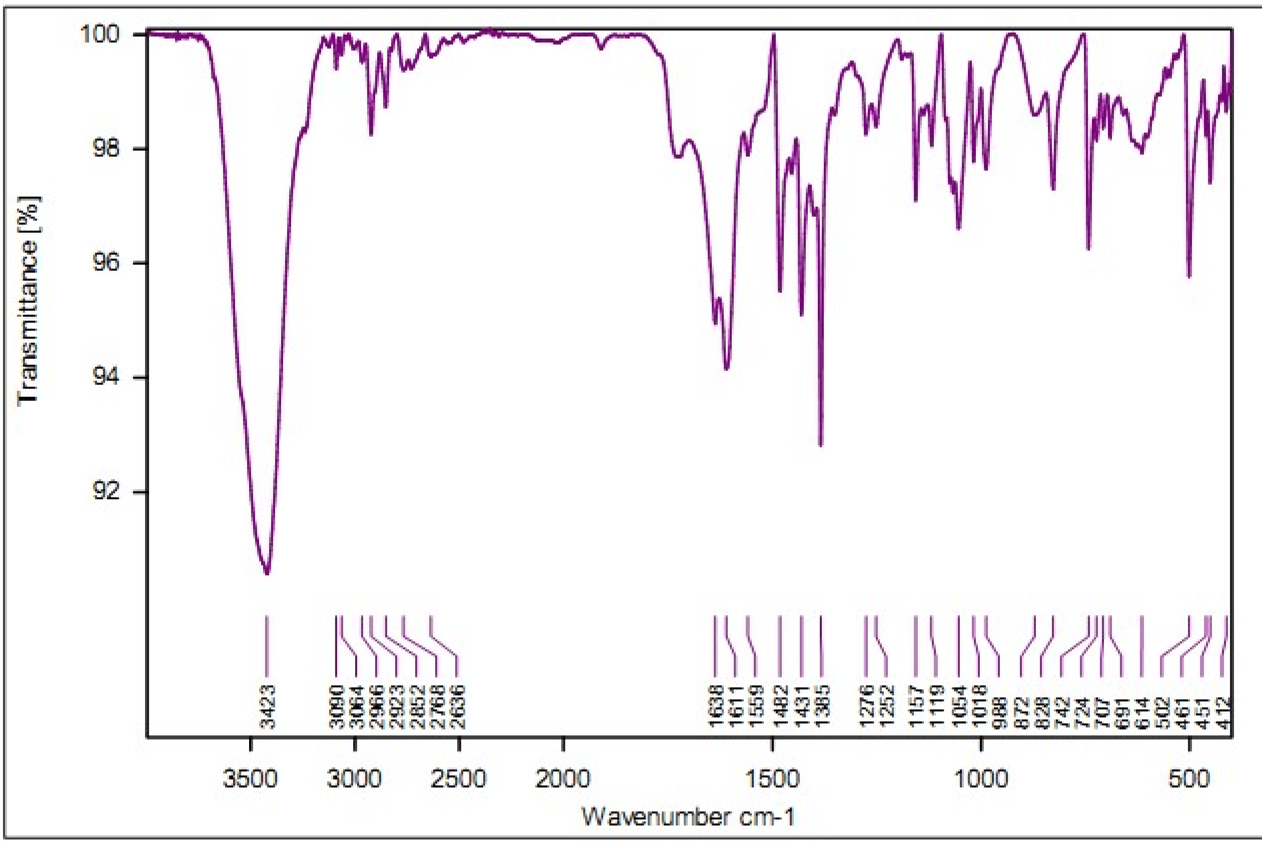

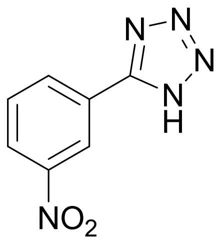

5-(3-nitrophenyl)-1H-tetrazole: ^{1}H NMR (400 MHz, DMSO): δ_H= 18.44 (br, 1H), 8.82 (s, 1H), 8.48-8.45 (d, *J*= 12 Hz, 1H), 8.43-8.39 (d of q, *J*(d)= 12 Hz, *J*(q)= 2 Hz, 1H), 7.93-7.87 (t, *J*= 12 Hz, 1H) ppm.

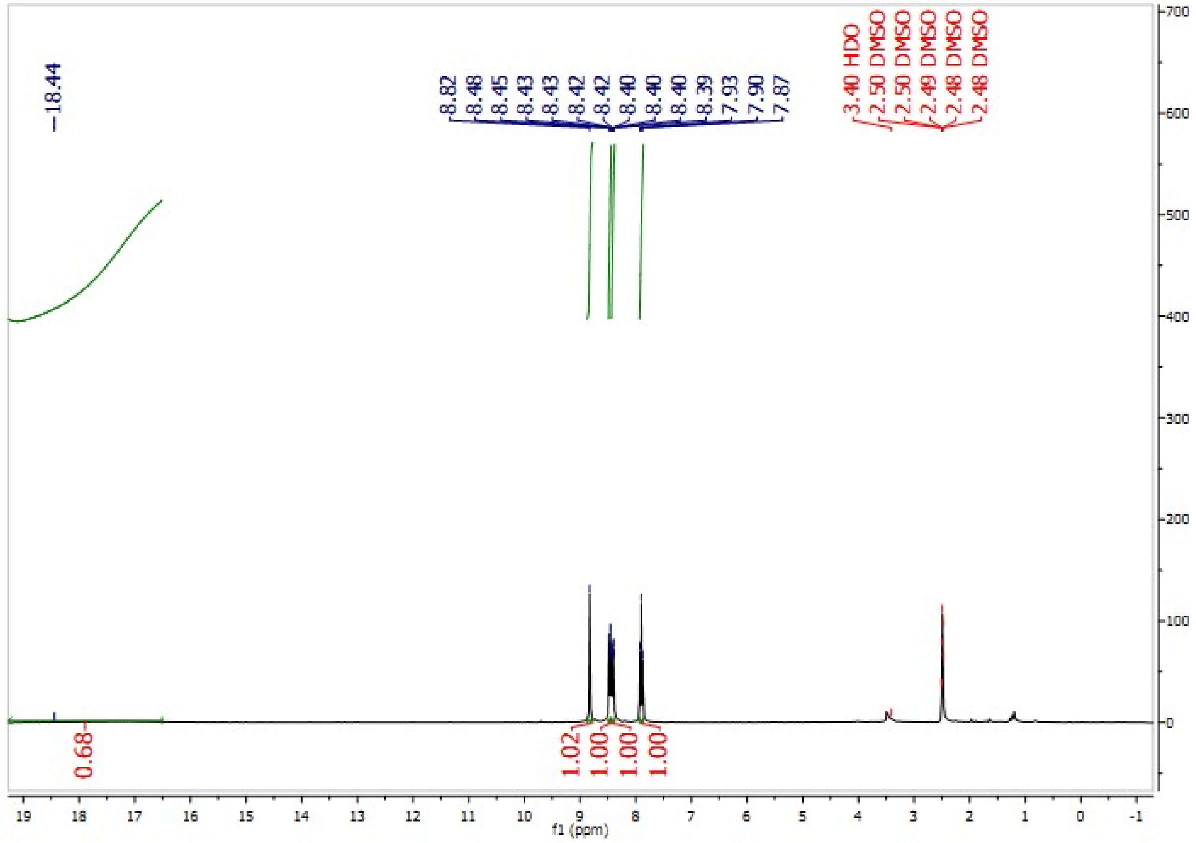

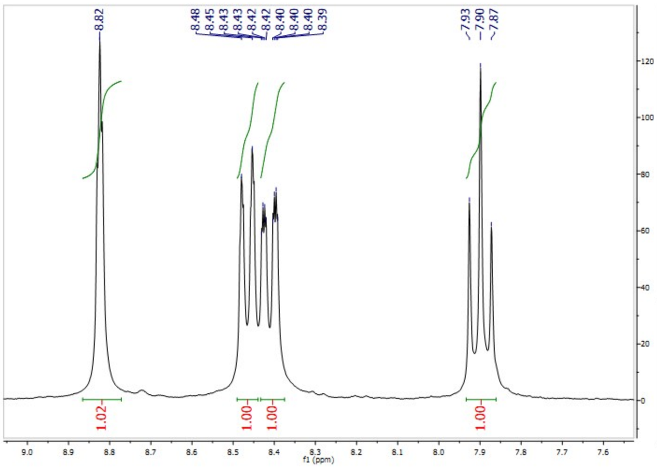

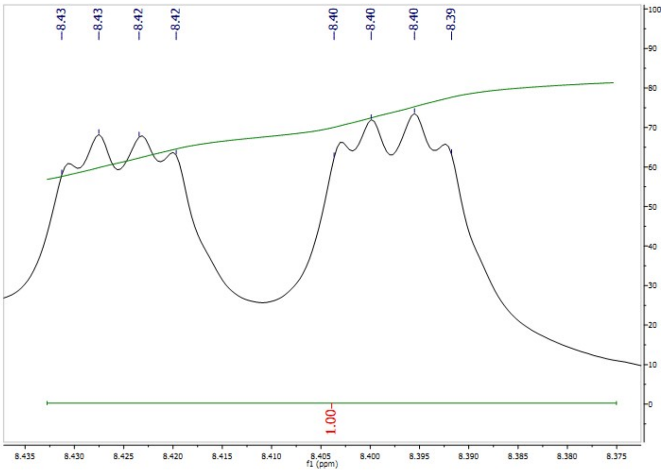

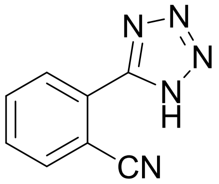

2-(1H-tetrazol-5-yl)benzonitrile: ^{1}H NMR (400 MHz, DMSO): δ_H= 8.10-8.06 (t, *J*= 8 Hz, 2H), 7.95-7.89 (t, *J*= 12 Hz, 1H), 7.80-7.75 (t, *J*= 12 Hz, 1H) ppm.

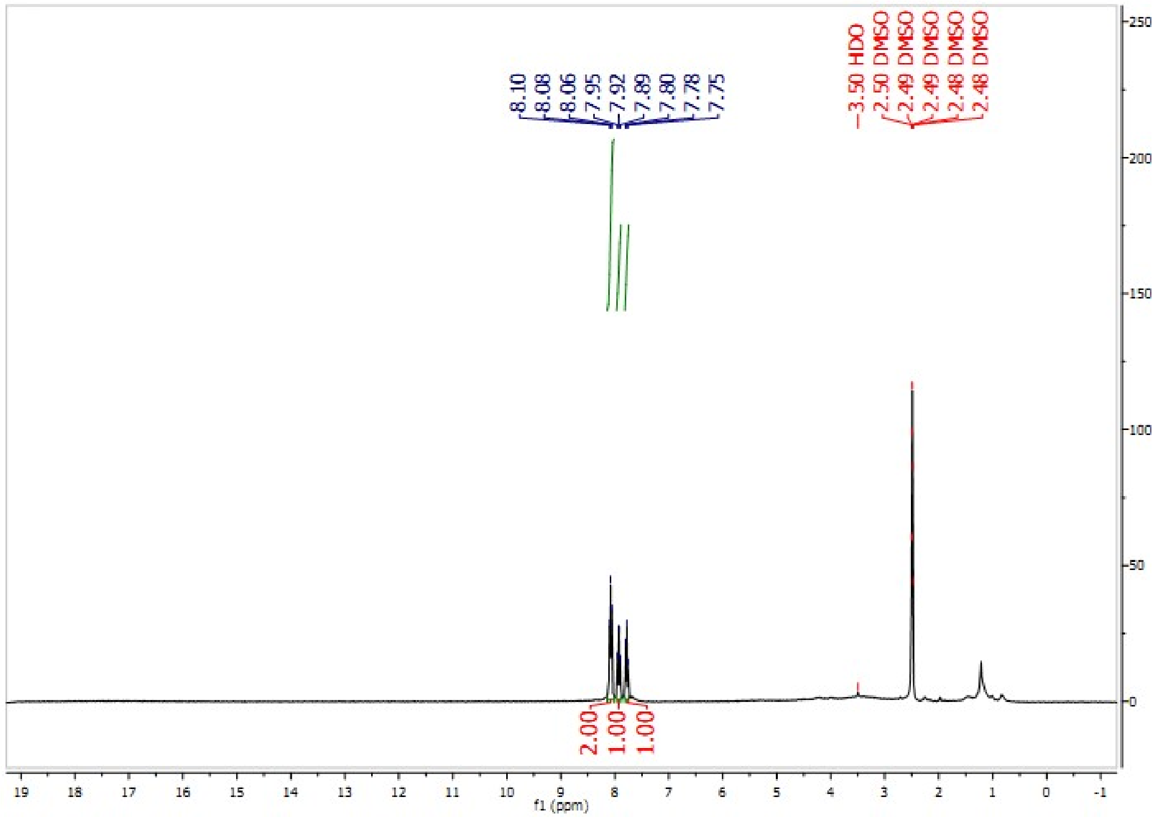

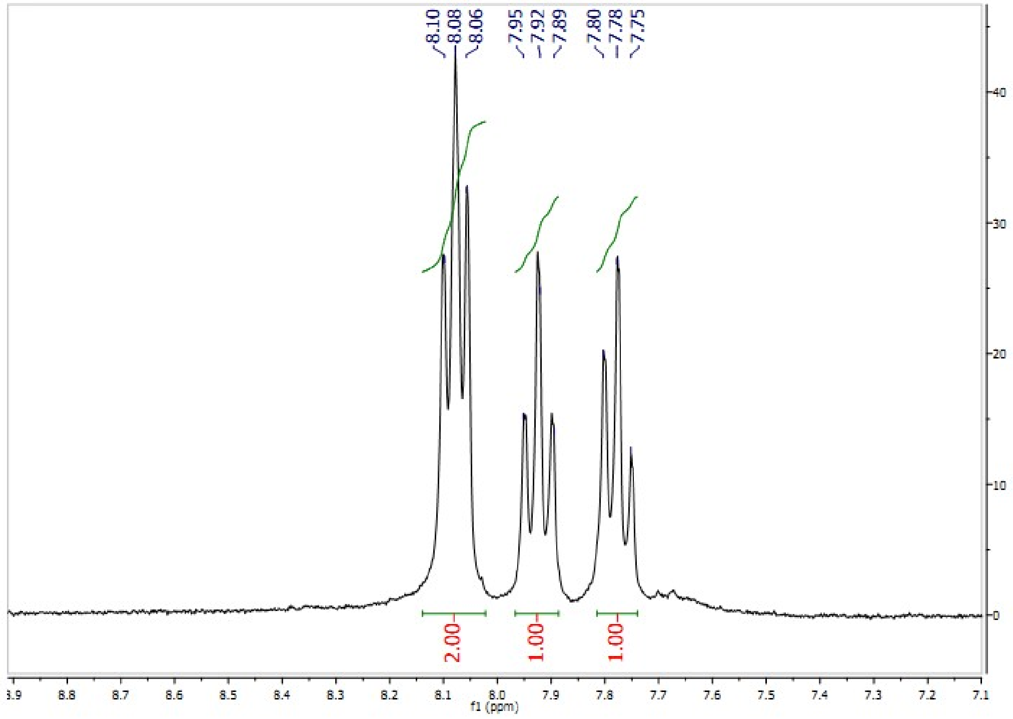

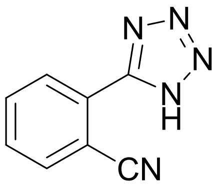

2-(1H-tetrazol-5-yl)benzonitrile: IR (KBr) cm^{-1}: 3426, 3082, 3029, 2922, 2582, 2721, 2615, 2229, 2005, 1735, 1638, 1609, 1581, 1493, 1454, 1408, 1384, 1280, 1242, 1199,1167, 1122, 1100, 1066, 1047, 1113, 999, 970, 856, 783, 756, 724, 705, 665, 585, 555, 518, 500.

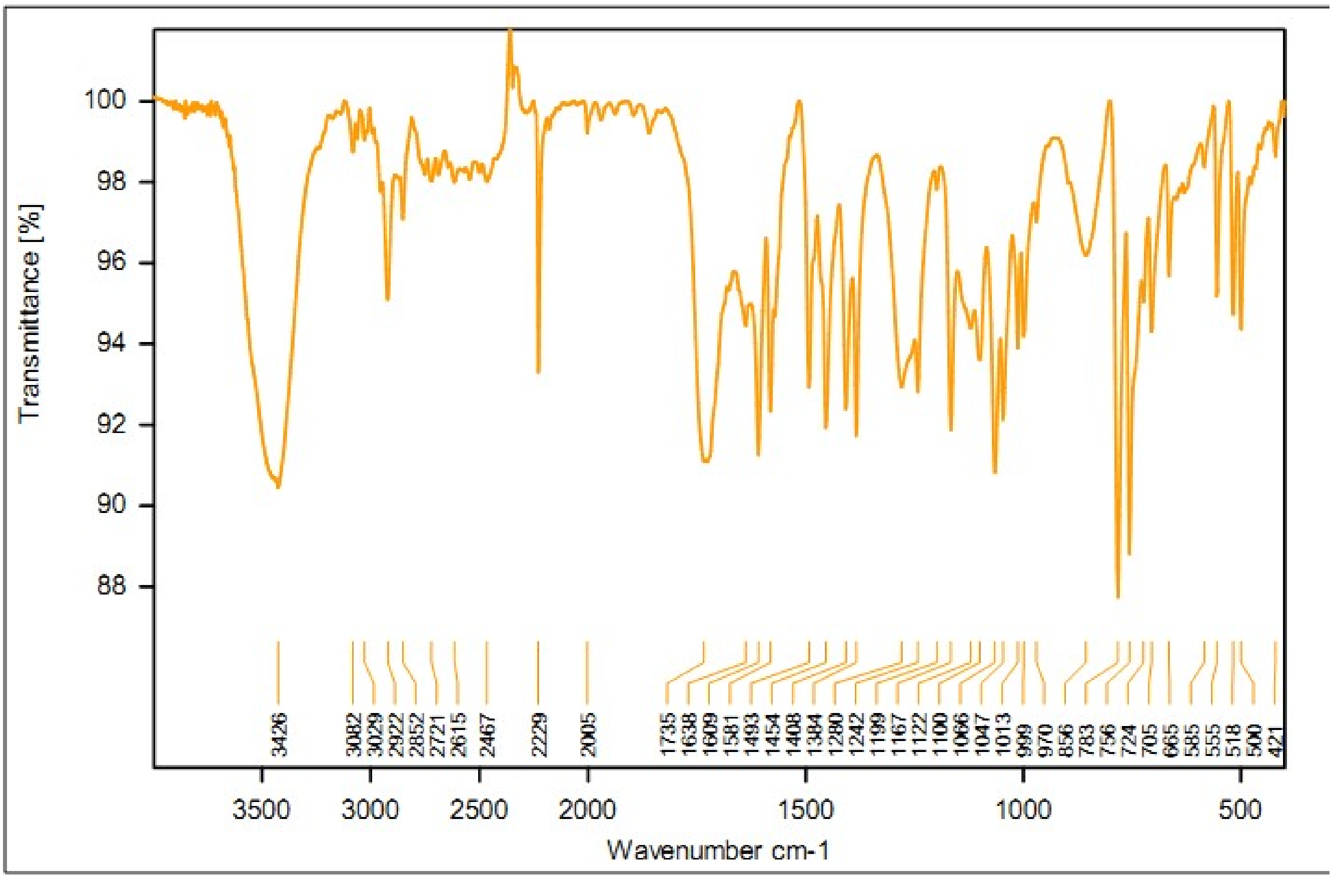

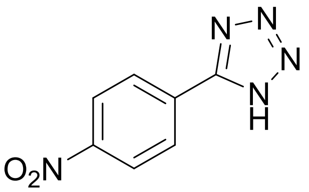

5-(4-nitrophenyl)-1H-tetrazole: ^{1}H NMR (400 MHz, DMSO): δ_H= 16.55 (br, 1H), 8.46-8.43 (d, *J*= 12 Hz, 2H), 8.31-8.28 (d, *J*= 12 Hz, 2H) ppm.

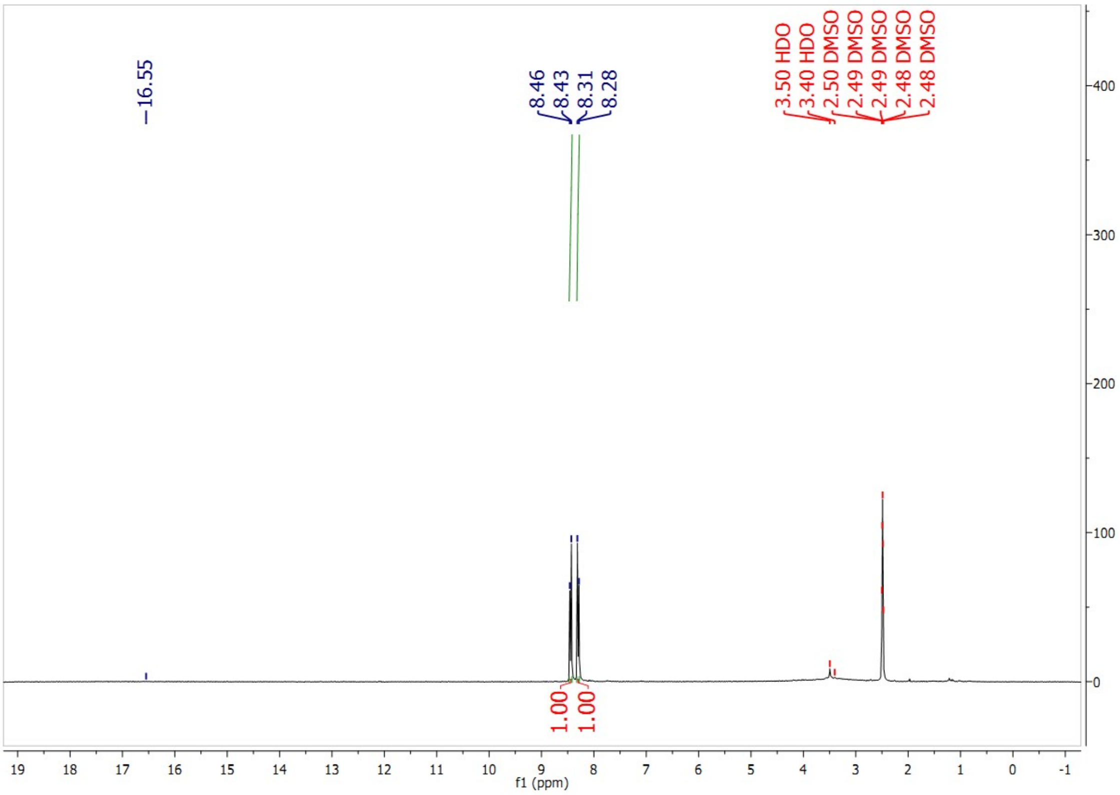

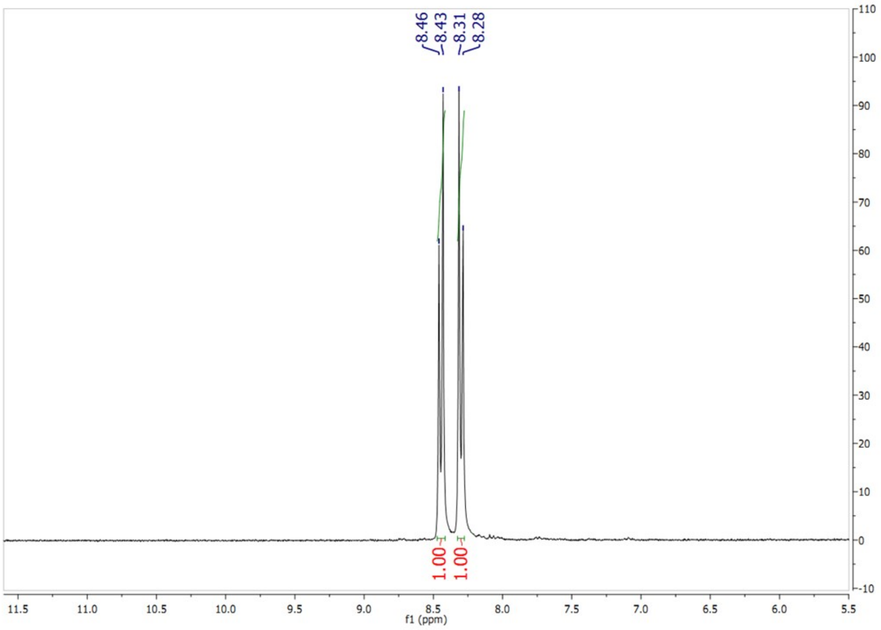

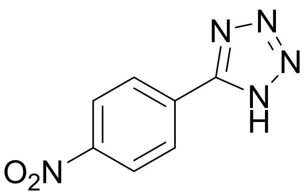

5-(4-nitrophenyl)-1H-tetrazole: IR (KBr) cm^{-1}: 3212, 3069, 2903, 1957, 1822, 1717, 1604, 1512, 1337, 1108, 1059, 991, 857, 773, 691, 491, 443.

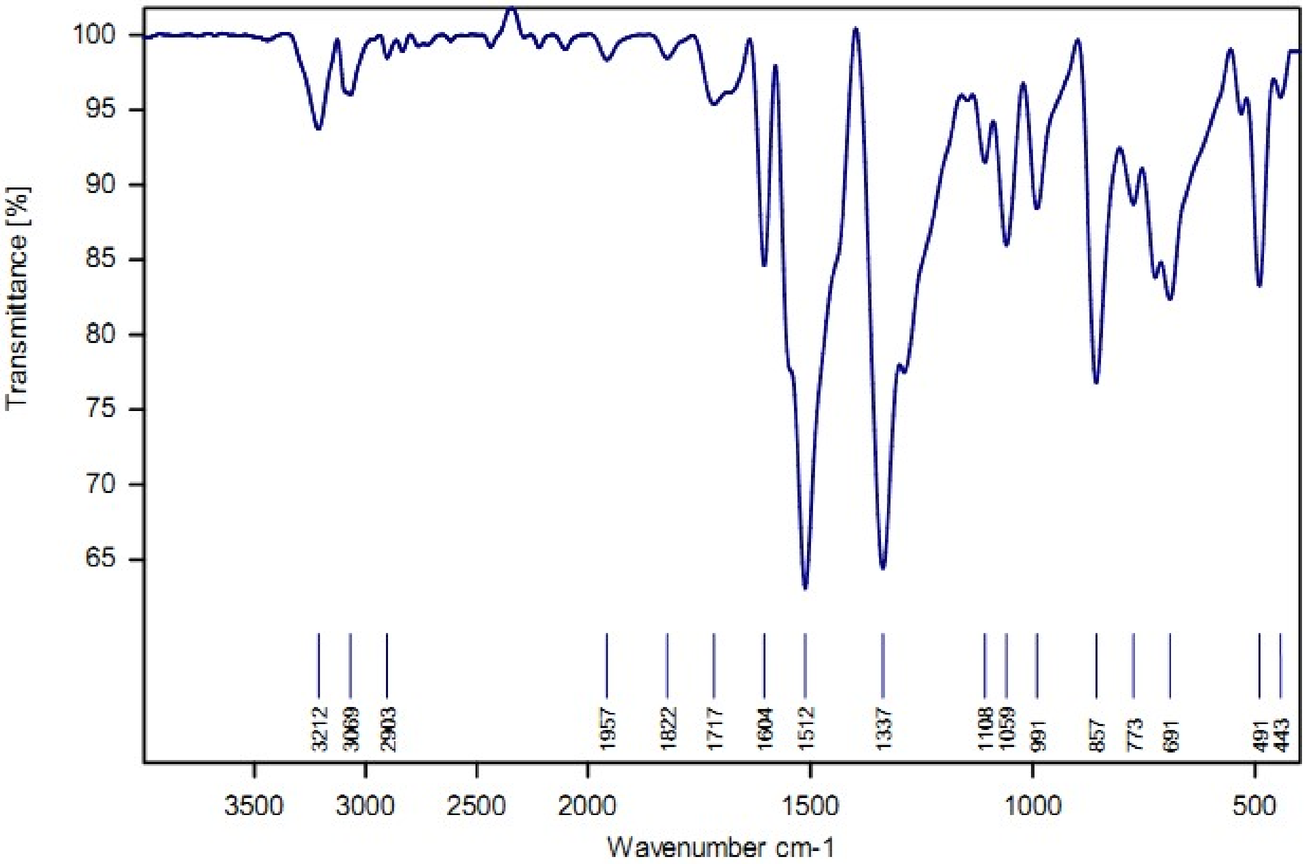

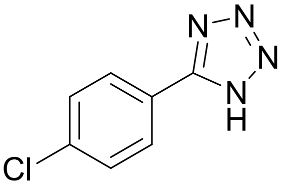

5-(4-chlorophenyl)-1H-tetrazole: ^{1}H NMR (400 MHz, DMSO): δ_H= 16.99 (br, 1H), 8.06-8.03 (d, *J*= 12 Hz, 2H), 7.71-7.68 (d, *J*= 12 Hz, 2H) ppm.

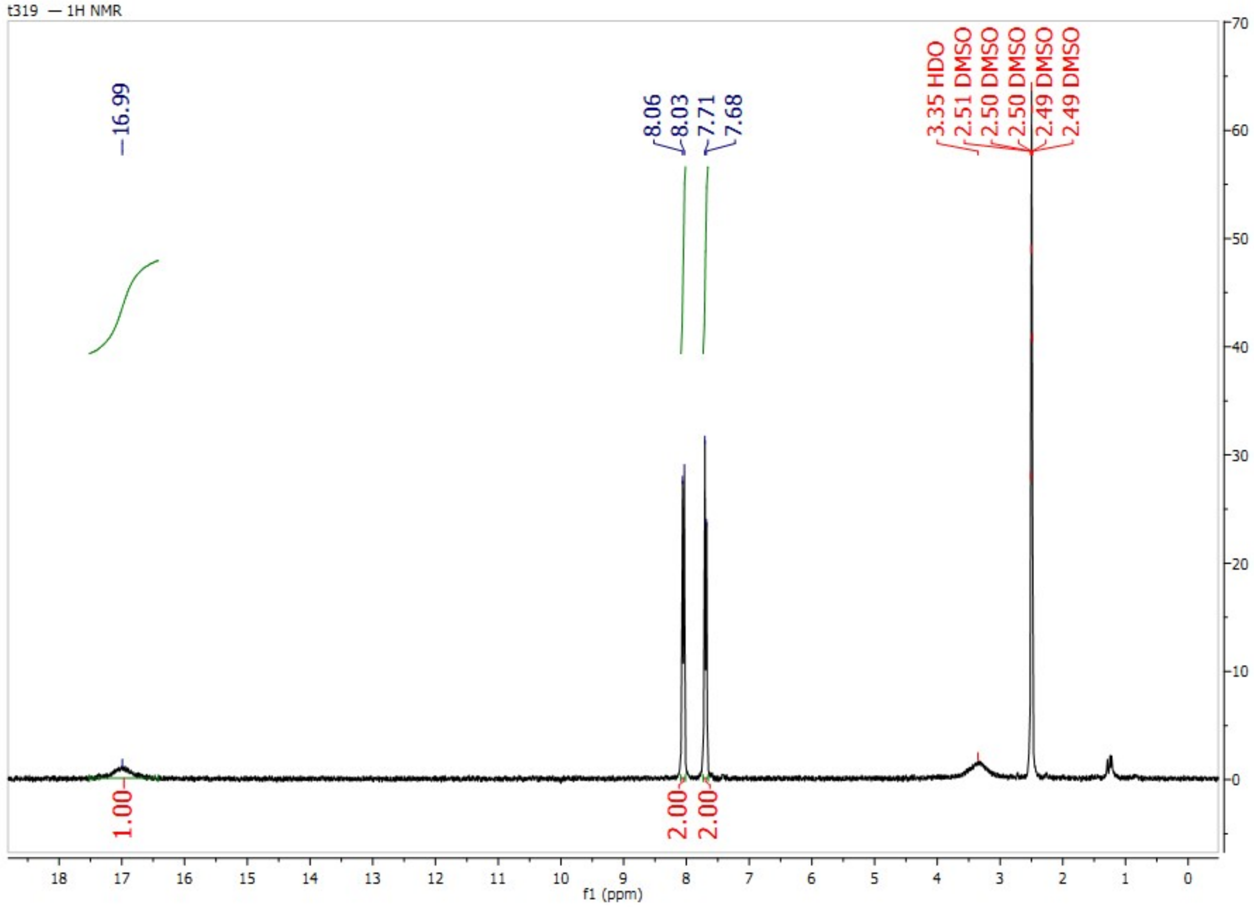

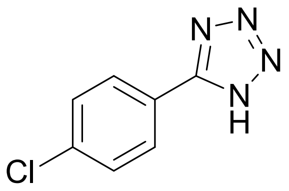

5-(4-chlorophenyl)-1H-tetrazole: IR (KBr) cm^{-1}: 3420, 3096, 3070, 2922, 2852, 2733, 1638, 1611, 1487, 1458, 1434, 1385, 1277, 1254, 1160, 1121, 1096, 1052, 1019, 988, 878, 829, 743, 709, 693, 623, 506, 464.

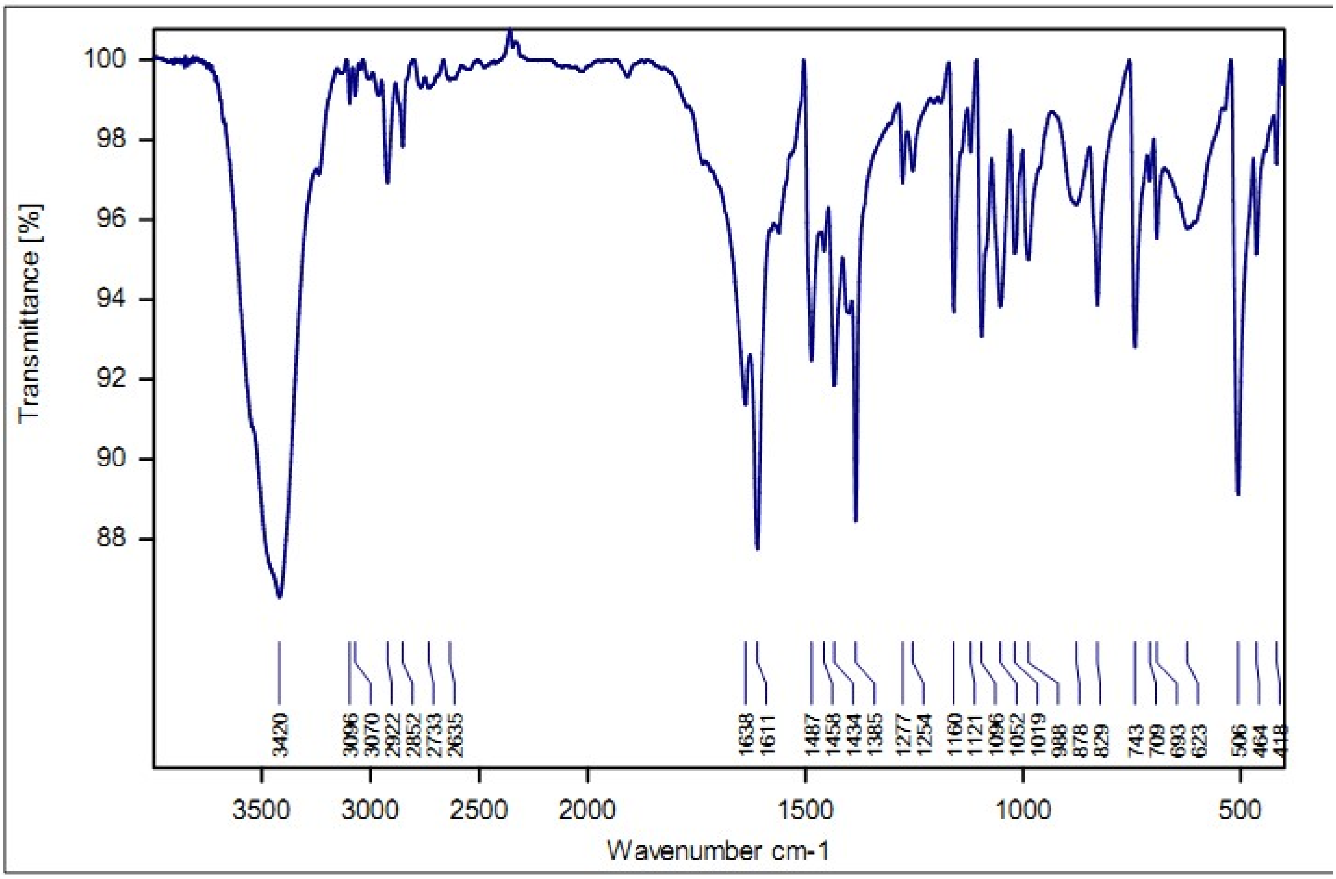

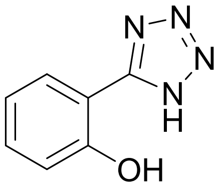

2-(1H-tetrazol-5-yl)phenol: ^{1}H NMR (400 MHz, DMSO): δ_H= 8.00-7.98 (d, *J*= 12 Hz, 1H), 7.43-7.38 (t, *J*= 8 Hz, 1H), 7.08-7.06 (d, *J*= 8 Hz, 1H), 7.03-6.97 (t, *J*= 12 Hz, 1H) ppm.

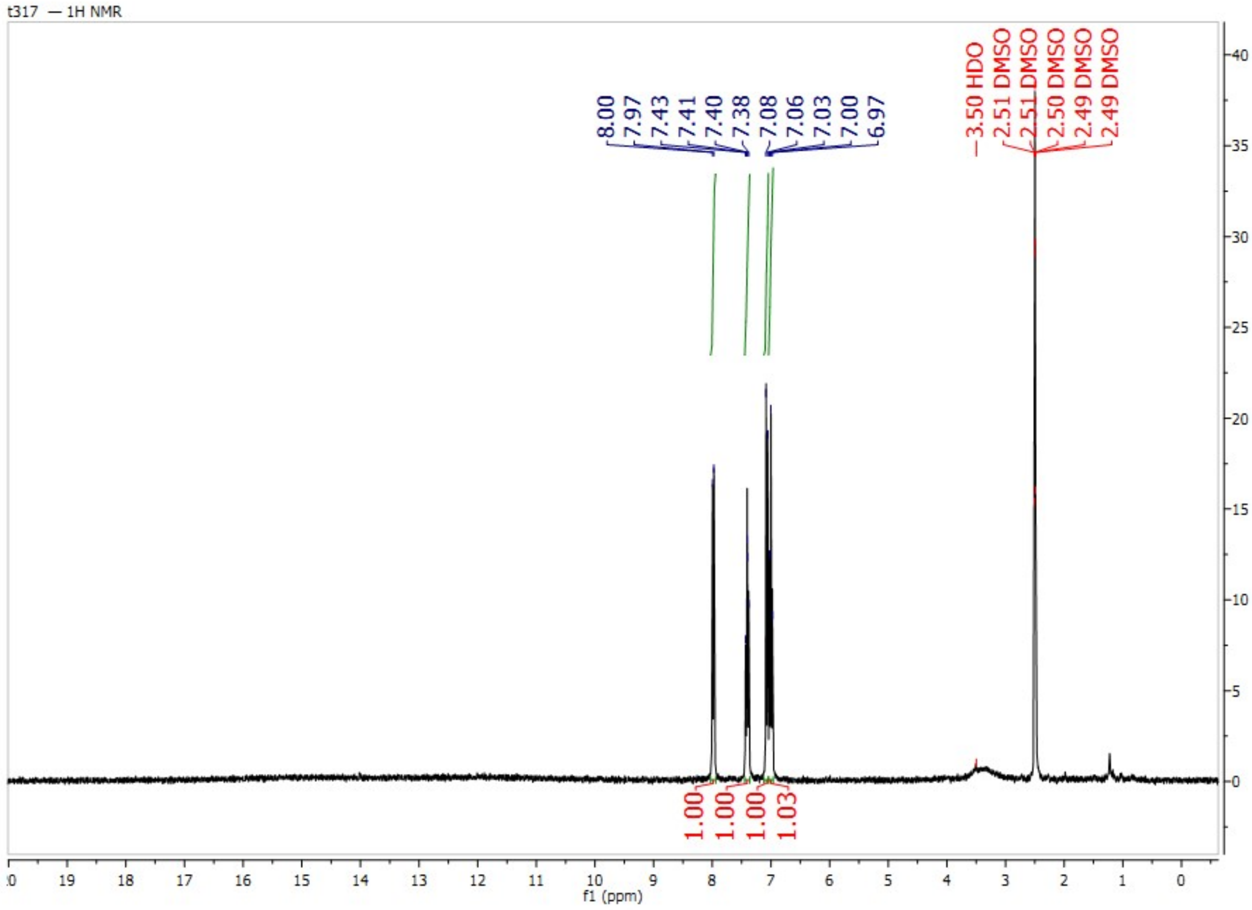

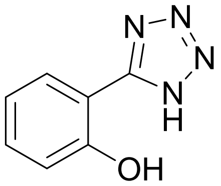

2-(1H-tetrazol-5-yl)phenol: IR (KBr) cm^{-1}: 3431, 3252, 3062, 2930, 2686, 2567, 1737, 1610, 1546, 1477, 1391, 1356, 1295, 1232, 1156, 1112, 1086, 1002, 941, 809, 741, 542.

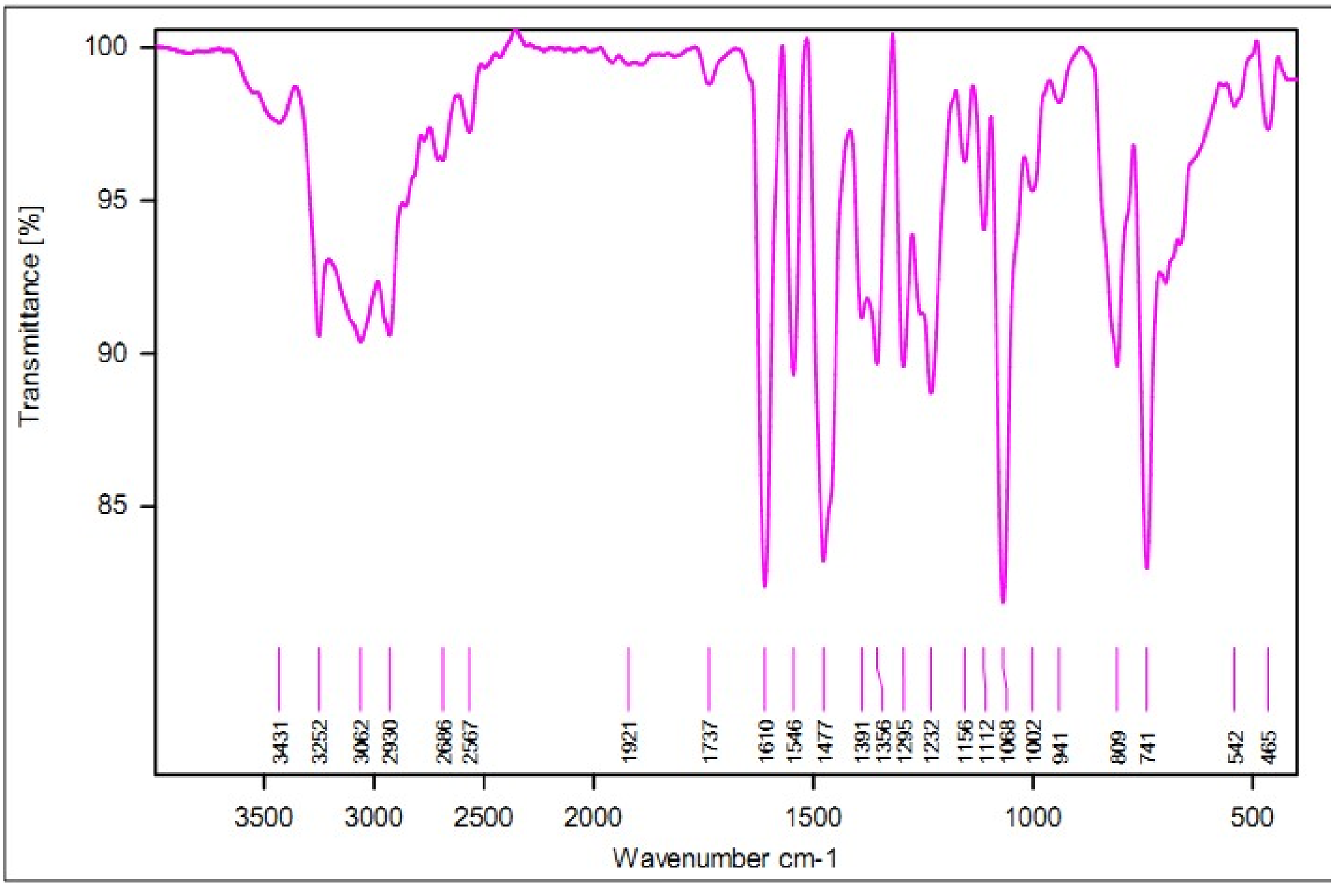

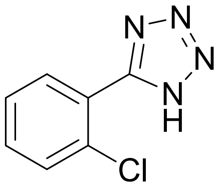

5-(2-chlorophenyl)-1H-tetrazole: ^{1}H NMR (400 MHz, DMSO): δ_H= 16.95 (br, 1H), 7.82-7.80 (d, *J*= 8 Hz, 1H), 7.73-7.70 (d, *J*= 12 Hz, 1H), 7.66-7.61 (t, *J*= 8 Hz, 1H), 7.58-7.54 (t, *J*= 8 Hz, 1H) ppm.

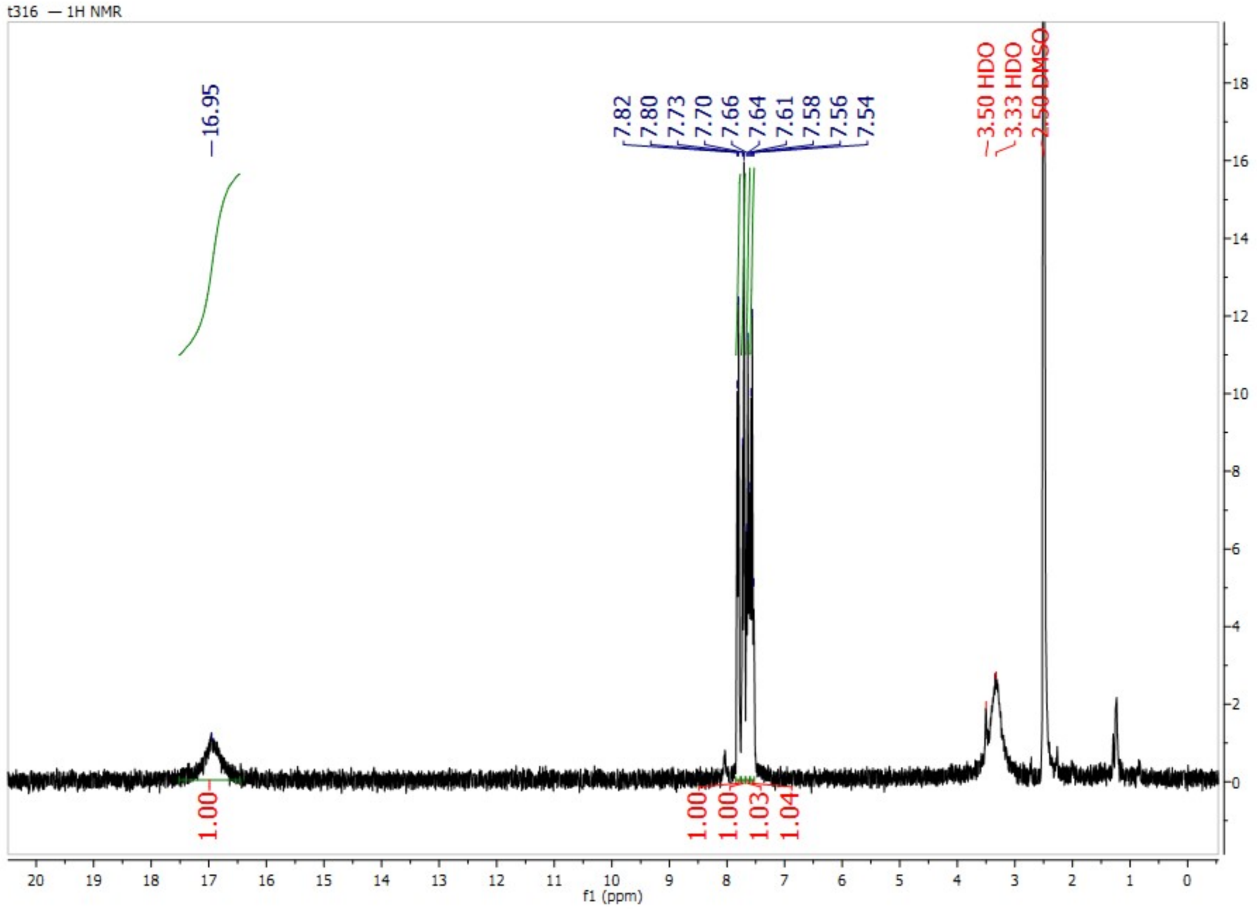

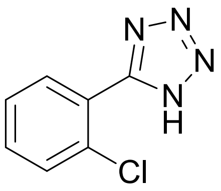

5-(2-chlorophenyl)-1H-tetrazole: IR (KBr) cm^{-1}: 3418, 2823, 2708, 1660, 1651, 1634, 1602, 1565, 1556, 1539, 1471, 1441, 1409, 1385, 1369, 1243, 1163, 1129, 1076, 1060, 1039, 1020, 1007, 988, 943, 874, 777, 747, 732, 651, 486, 453, 434.

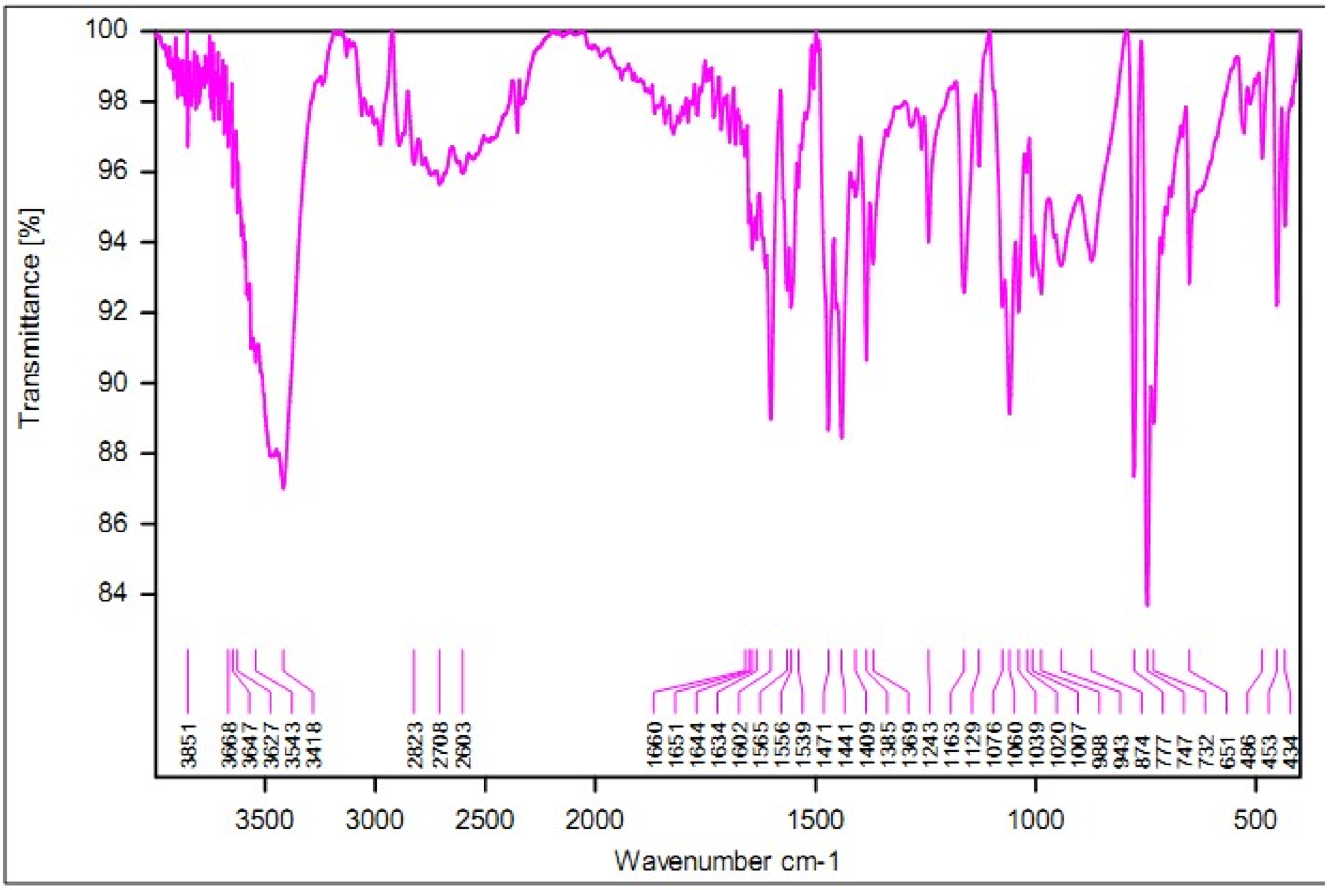

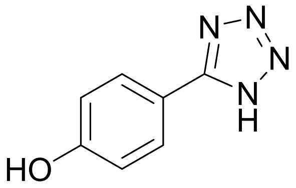

4-(1H-tetrazol-5-yl)phenol: ^{1}H NMR (400 MHz, DMSO): δ_H= 16.51 (br, 1H), 10.15 (br, 1H), 7.87-7.84 (d, *J*= 12 Hz, 2H), 6.96-6.93 (d, *J*= 12 Hz, 1H) ppm.
IR (KBr) cm^{-1}:

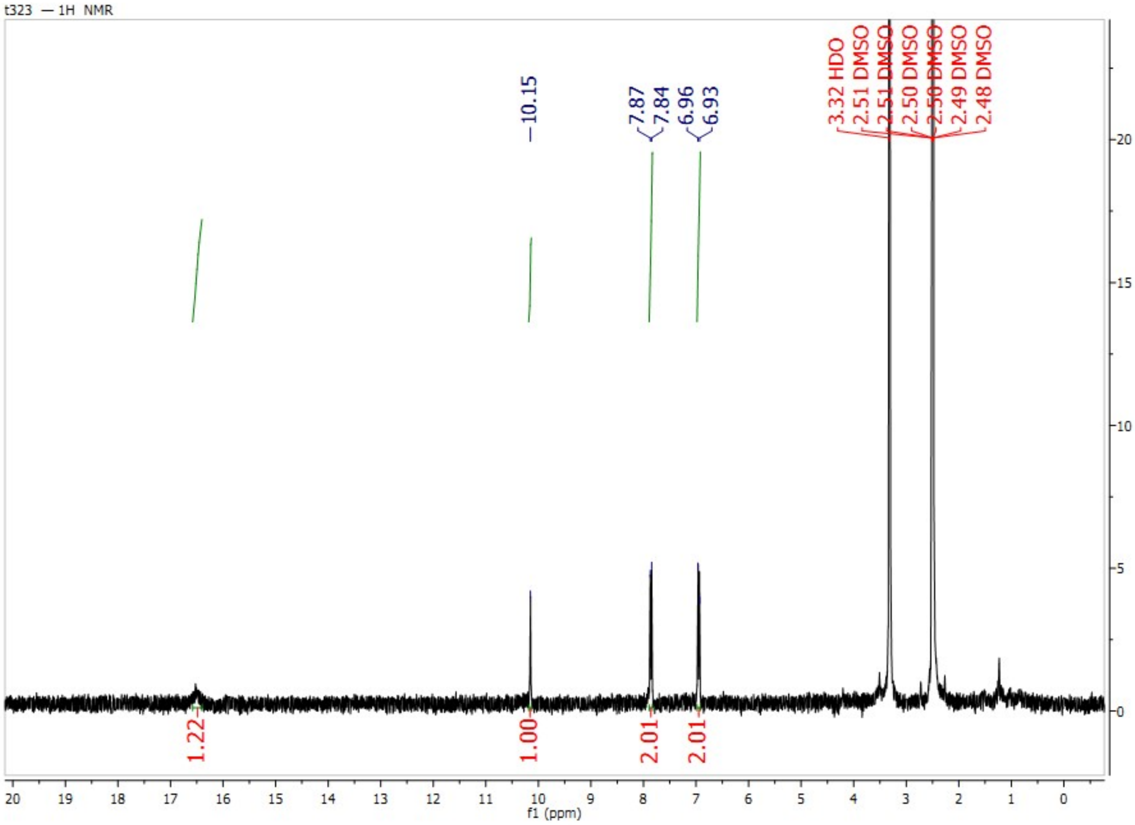

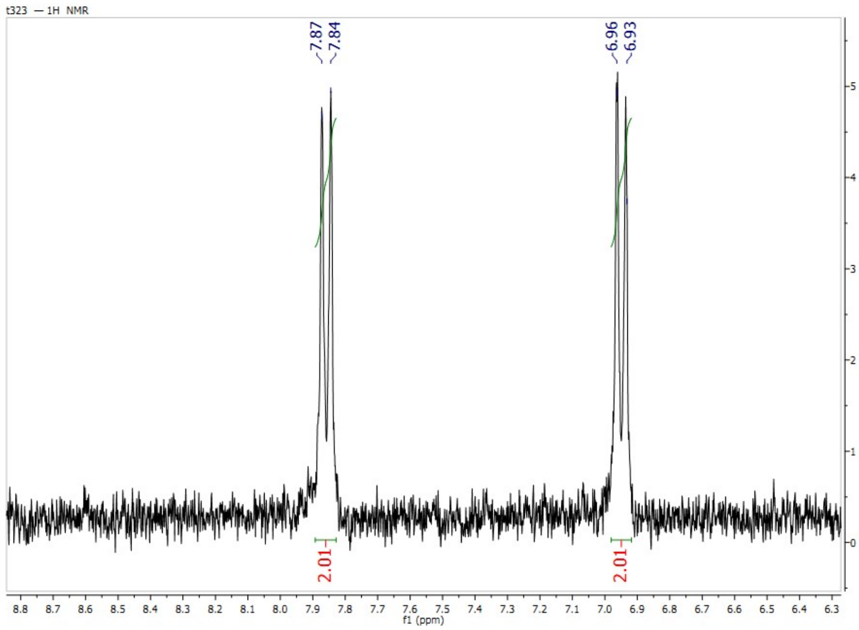

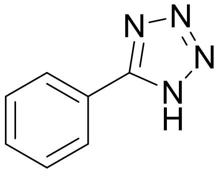

5-phenyl-1H-tetrazole: ^{1}H NMR (400 MHz, DMSO): δ_H= 16.88 (br, 1H), 8.06-8.03 (m, 2H), 7.63-7.59 (m, 3H) ppm.

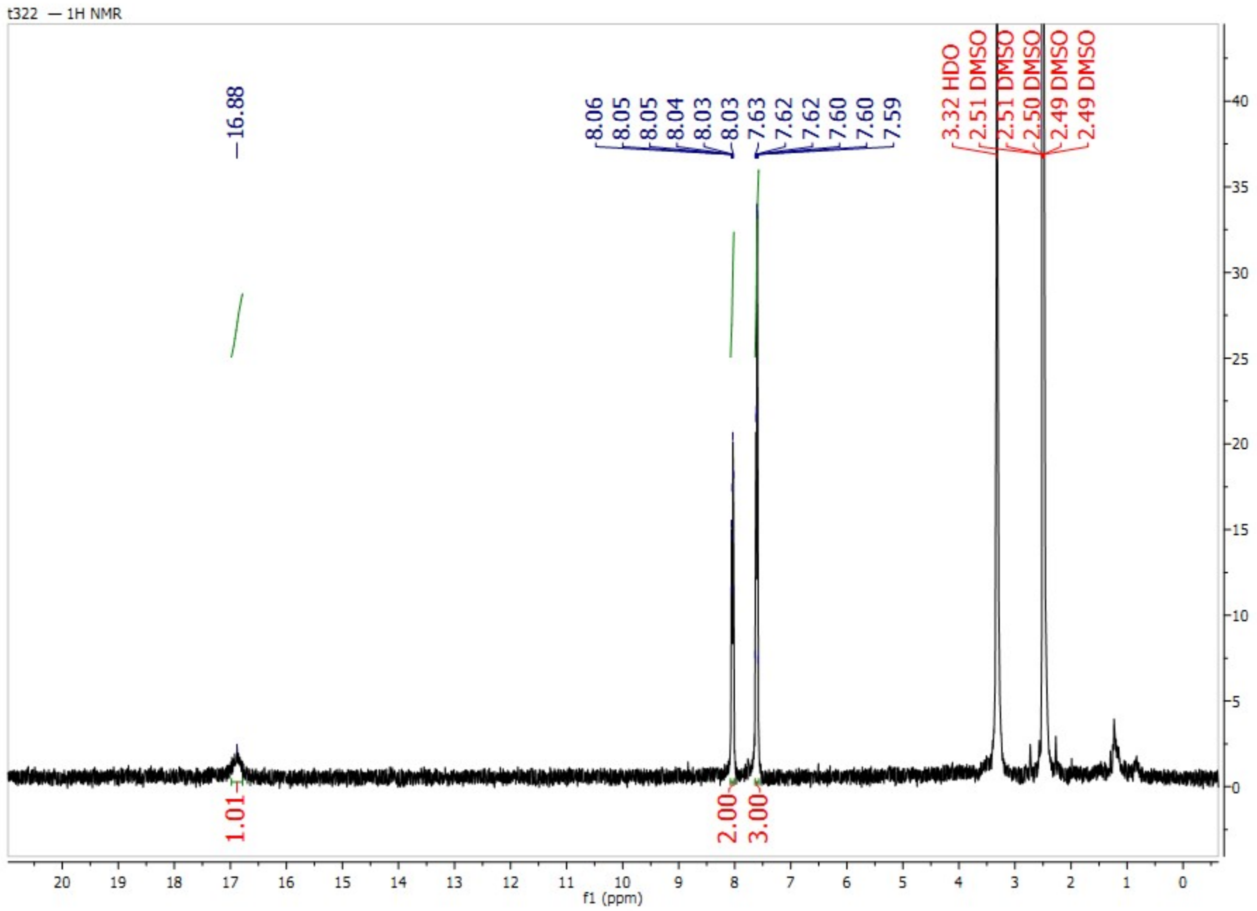

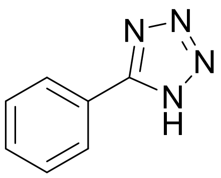

5-phenyl-1H-tetrazole: IR (KBr) cm^{-1}: 3423, 3055, 2985, 2901, 2832, 1813, 1638, 1611, 1562, 1486, 1466, 1411, 1384, 1288, 1256, 1163, 18084, 1057, 1034, 1015, 991, 959, 925, 784, 726, 703, 687, 619, 493, 462.

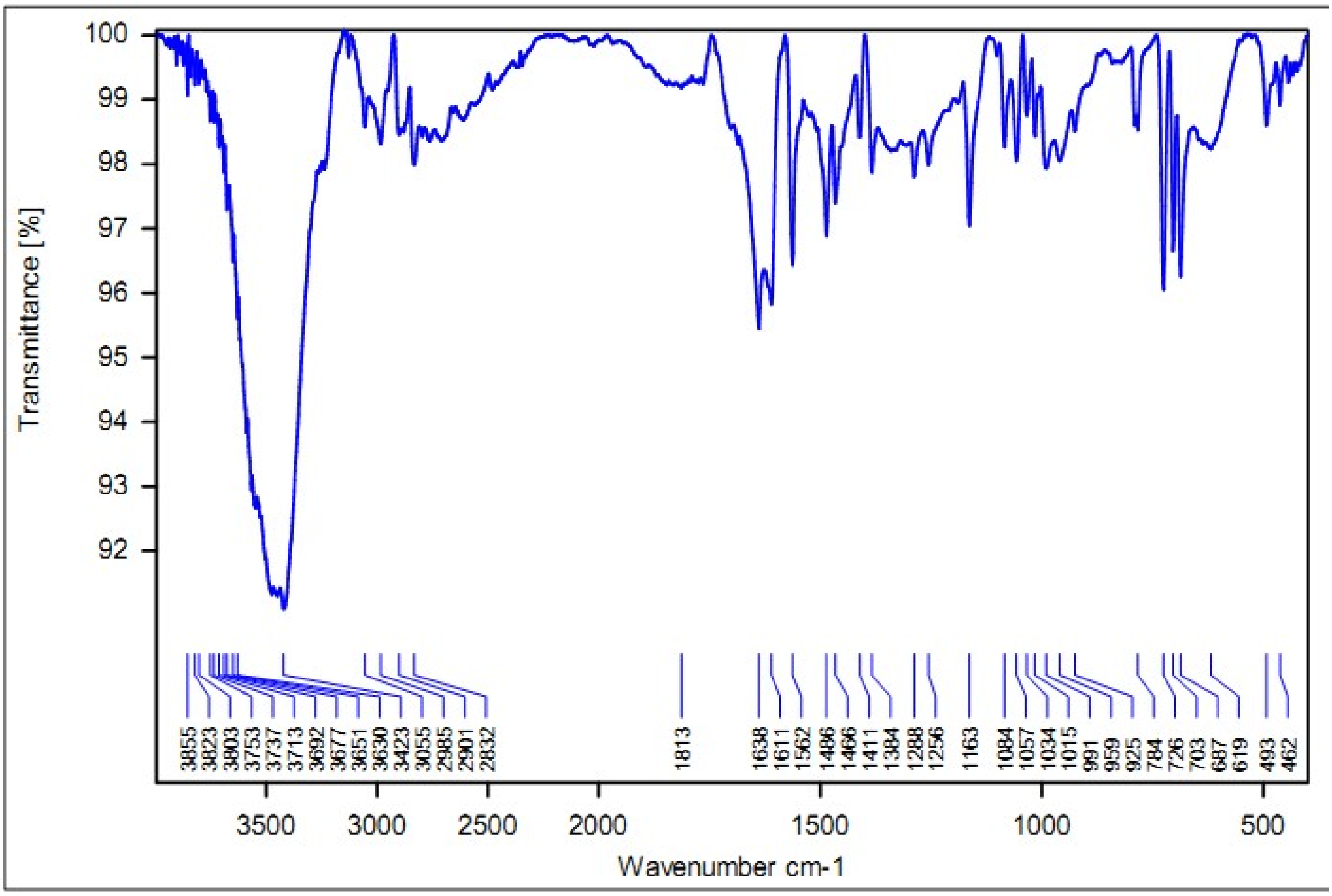

Transmittance [%]
Wavenumber cm-1
